# Tunable Assembly of Photocatalytic Colloidal Coatings for Antibacterial Applications

**DOI:** 10.1021/acsapm.4c01436

**Published:** 2024-08-23

**Authors:** Constantina Sofroniou, Alberto Scacchi, Huyen Le, Edgar Espinosa Rodriguez, Franck D’Agosto, Muriel Lansalot, Patrick S. M. Dunlop, Nigel G. Ternan, Ignacio Martín-Fabiani

**Affiliations:** † Department of Materials, Loughborough University, Loughborough LE11 3TU, United Kingdom; ‡ Department of Applied Physics, Aalto University, P.O. Box 11000, Aalto FI-00076, Finland; § Department of Bioproducts and Biosystems, Aalto University, P.O. Box 16100, Aalto FI-00076, Finland; ∥ Universite Claude Bernard Lyon 1, CPE Lyon, CNRS, UMR 5128, Catalysis, Polymerization, Processes and Materials (CP2M), Villeurbanne F-69616, France; ⊥ Nanotechnology and Integrated BioEngineering Centre (NIBEC), Ulster University, Newtownabbey BT37 0QB, Northern Ireland, United Kingdom; # Nutrition Innovation Centre for Food and Health (NICHE), Ulster University, Coleraine, Londonderry BT52 1SA, Northern Ireland, United Kingdom; △ Department of Mechanical and Materials Engineering, University of Turku, Turku 20500, Finland

**Keywords:** colloids, coatings, photocatalysis, self-assembly, stratification, antibacterial, titanium dioxide

## Abstract

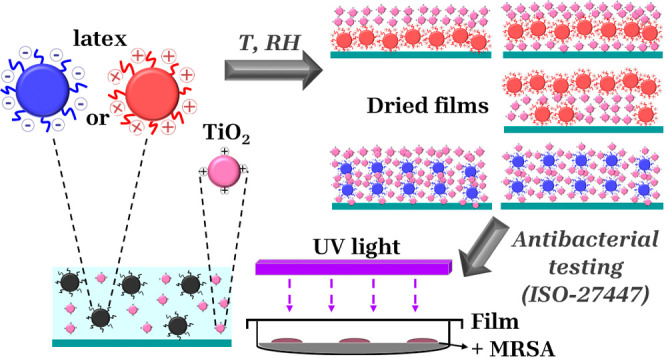

In this study, evaporation-induced size segregation and interparticle interactions are harnessed to tune the microstructure of photocatalytic colloidal coatings containing TiO_2_ nanoparticles and polymer particles. This enabled the fabrication of a library of five distinct microstructures: TiO_2_-on-top stratification, a thin top layer of polymer or TiO_2_, homogeneous films of raspberry particles, and a sandwich structure. The photocatalytic and antibacterial activities of the coatings were evaluated by testing the viability of Methicillin-resistant *Staphylococcus aureus* (MRSA) bacteria using the ISO-27447 protocol, showing a strong correlation with the microstructure. UVA irradiation for 4 h induces a reduction in MRSA viability in all coating systems, ranging from 0.6 to 1.1 log. Films with TiO_2_-enriched top surfaces exhibit better resistance to prolonged exposure to disinfection and bacterial testing. The remaining systems, nonetheless, present higher antibacterial activity because of a larger number of pores and coating defects that enhance light and water accessibility for the generation and transport of reactive oxygen species. This work establishes design rules for photocatalytic coatings based on the interplay between performance and film architecture, offering valuable insights for several applications, including antibacterial surfaces, self-cleaning/antifogging applications, and water purification.

## Introduction

Antimicrobial resistance, which occurs when microorganisms such as bacteria, viruses, or fungi stop responding to drugs designed to inhibit or eradicate them, is one of the biggest challenges to public health.^1^ Nowadays, healthcare-associated infections constitute the second most common source of death,^2^ with the majority of these caused by bacteria.^3^ Besides direct contact with an infected person, a key pathway for bacterial transmission is through contact with a contaminated surface. Indeed, cross-contamination through the hands of medical personnel contributes to 20–40% of infections within a hospital environment.^4^ The current strategy to prevent cross-transmission is to have in place hand cleaning protocols. However, this approach has been proven to be inefficient on its own, highlighting the need of other supportive measures.^5^

One of these measures involves the fabrication and use of antimicrobial surfaces and coatings.^6−9^ An antibacterial coating can act by killing bacteria (bactericidal), preventing biofilm formation (antifouling),^10^ or both. Bactericidal activity can be achieved via the release of biocides from the coating, e.g., silver, copper, or zinc ions. These ions can disrupt bacterial cell membranes, interfere with cellular processes, and ultimately lead to cell death. Silver ions, released from either silver nanoparticles or continuous metallic layers, have been extensively used in coatings as biocides.^11,12^ Vasilev and co-workers have been one of the first teams to work on silver nanoparticles in coatings for medical devices,^13^ raising the concern of silver materials being potentially harmful to tissues and organs.^14^ Other bactericidal mechanisms are contact killing by means of quaternary ammonium compounds (QACs)^15,16^ or the use of spikey surface textures that physically damage cells.^17−21^

Coatings can incorporate photocatalytic materials able to produce reactive oxygen species (ROS) when exposed to light, which can lead to degradation of bacterial cell membranes, proteins, and DNA.^22^ Titanium dioxide (TiO_2_), usually in its anatase phase, is the most commonly studied photocatalyst. It has been used in a plethora of applications, including water purification,^23,24^ air treatment,^25,26^ foodstuffs,^27,28^ and self-cleaning surfaces.^29^ However, when incorporated into coatings, the ROS generated by titanium dioxide can result in degradation of the organic binder.^30^ Moreover, the production of TiO_2_ is associated with a high carbon footprint, primarily due to the energy-intensive processes involved.^31,32^ Therefore, to successfully introduce photocatalytic titanium dioxide into coatings, it is crucial to both minimize the usage of TiO_2_ and find ways to reduce the level of binder degradation.

An approach that might mitigate both issues is self-stratification. This term refers to the phenomenon in which two or more colloidal species segregate by size in the direction perpendicular to the substrate as the solvent evaporates.^33^ This process enables different coating microstructures to be achieved depending on parameters such as the wet film thickness (*H*), particle size ratio, temperature (*T*), relative humidity (RH), and particle volume fraction (φ). Small-on-top and large-on-top stratification architectures have been reported, both experimentally and theoretically, in binary colloidal mixtures upon drying.^34−37^ In rare cases, some more complex architectures have been reported such as small-large-small or large-small-large sandwich structures.^38^ Films showing a single layer of large particles trapped at the top film surface have also been reported experimentally^39^ and predicted by simulations.^40,41^ In all of the mentioned examples, the systems under study consisted of blends of charge-stabilized particles of the same sign. However, particle surface charge can play a key role, leading to raspberry complexes when repulsion between particle populations is not strong enough.^42^ Therefore, stratification can be harnessed to enable a more efficient utilization of additives by strategically placing them at the desired position within the dried coating, e.g., a biocide at the top of the film to maximize contact with microorganisms. We reported the first example of harnessing stratification in the fabrication of antibacterial coatings, where bactericidal zinc oxide (ZnO) nanoparticles were driven to the top of coatings, enhancing their antibacterial efficiency.^34^ However, the use of size segregation processes in the assembly of photocatalytic coatings remains unexplored, to the best of our knowledge.

**Scheme 1:**
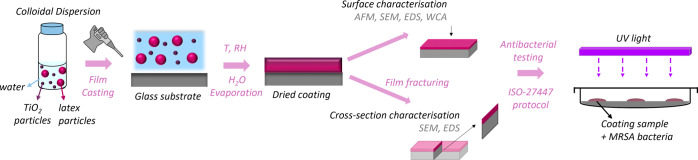
Graphical Representation of the Experimental Work Conducted in This Study A colloidal dispersion containing TiO_2_ and latex particles in water is prepared and cast onto glass substrates. The coatings are allowed to form under different temperature and relative humidity conditions. The resulting film surfaces and cross sections are then characterized using AFM, SEM, EDS, and WCA measurements. The antibacterial properties of the coatings are tested against MRSA bacteria following the ISO-27447 protocol.

Here, we report an experimental and computational study on TiO_2_/polymer particle films and their application as photocatalytic antibacterial coatings. We show how by tuning temperature or relative humidity, as well as the electrostatic interactions between small and large particles, the final film architecture can be tailored to obtain a range of microstructures. We demonstrate how the microstructure is strongly correlated with the photocatalytic and antibacterial activity against Methicillin-resistant *Staphylococcus aureus* (MRSA). The experimental strategy followed in this work is graphically summarized in Scheme 1. This study serves as a proof of concept for the use of size segregation and particle interactions in achieving on-demand photocatalytic properties, not only for antibacterial applications but also in other areas such as self-cleaning and antifogging surfaces.

## Materials and Methods

### Materials

Titanium(IV) oxide Aeroxide P25 in the form of a white powder (TiO_2_, Fisher Scientific) consists of both anatase and rutile phases, with a composition of 76.3 wt % anatase and 10.6 wt % rutile.^43^ Hydrochloric acid 37 wt % (Merck), isopropyl alcohol (IPA, Fisher Scientific, ≥99.8%), sodium 4-styrenesulfonate (SSNa, Aldrich, >99.5%), 2,2′-azobis(2-methylpropionamidine) dihydrochloride (AIBA, Aldrich, 97%), 4,4′-azobis(4-cyanopentanoic acid) (ACPA, Aldrich, >98%), 1,3,5-trioxane (Aldrich ≥99%), sodium bicarbonate (NaHCO_3_, Aldrich, 99.7%), methyl methacrylate (MMA, 99%), *n*-butyl acrylate (BA, Aldrich, 99%), and ammonium persulfate (APS, Acros Organics, 98%) were purchased and used as received. *N*-3-(Dimethylamino)propyl methacrylamide (DMAPMA, Aldrich, 99%) was purified by passing over a column of basic aluminum oxide. 4-Cyano-4-thiothiopropylsulfanyl pentanoic acid (CTPPA) was synthesized following a reported procedure.^44^ The synthesis of PDMAPMA and PSSNa macromolecular RAFT (macroRAFT) agents as well as the emulsion copolymerization of *n*-butyl acrylate and methyl methacrylate in the presence of these macroRAFT agents to form latex particles can be found in the Supporting Information (SI).

### Preparation of TiO_2_ Nanoparticle Dispersion

A 5 wt % stock dispersion of TiO_2_ was prepared by dispersing P25 powder in Milli-Q water. pH was adjusted to 3–3.5 with hydrochloric acid. The dispersion was mixed with a magnetic stirrer for 1 h and then sonicated for 15 min in an ultrasonic bath. To select only the small particles and avoid collecting aggregates, the TiO_2_ dispersion was then centrifuged (Heraeus Labofuge 400R centrifuge, Thermo Scientific) at 4000 rpm for 1 h and 15 min in 15 mL Falcon tubes, and the supernatant was carefully collected. The solid content of the supernatant was determined to be 0.1 wt %, as per thermogravimetric analysis (Discovery TGA 550, TA Instruments). A water evaporation step followed, where the supernatant was stirred with a magnetic bar on a hot plate at 100 °C until a final TiO_2_ solid content of 2 wt % was reached. The positively charged TiO_2_ nanoparticles in the 2 wt % dispersion, of about 40 nm size, were stable for several weeks.

### Film Formation

The 2 wt % TiO_2_ dispersion was sonicated for 10 min prior to mixing with latex dispersions to minimize aggregation. The appropriate amounts of TiO_2_ and latex (either positively or negatively charged) were then mixed in Milli-Q water (adjusted pH at 3–3.5) and the blend was vortexed for a few seconds. The final solids content (TiO_2_ and latex particles excluding water) was 3 wt %. 400 μL of the blend were then cast on square glass coverslips (18 mm × 18 mm), previously cleaned with an ultraviolet (UV) ozone cleaner (Ossila, Sheffield) for 10 min. Blends were left to dry in slow evaporation conditions (ambient laboratory conditions, 21 ± 1 °C, RH 50 ± 5%) over a 4–5 h period or in fast evaporation conditions (60 ± 1 °C, RH 10 ± 5%) in an oven (OVA031, Gallenkamp) for 30 min. The final film thickness was approximately 20 μm.

### Dynamic and Electrophoretic Light Scattering (DLS-ELS)

Particle diameter, size distribution, and ζ-potential measurements were carried out using a Zetasizer Ultra (Malvern Panalytical, Malvern, U.K.). The samples (TiO_2_, latex dispersion, or blend) were diluted to 0.1 wt %. All of the measurements were carried out at 25 °C in folded capillary cells (DTS1070), and three repetitions were performed for each sample.

### Atomic Force Microscopy (AFM)

AFM topography and adhesion measurements were performed with a Bruker BioScope Resolve instrument (Bruker, Santa Barbara, CA). All of the measurements were performed in the PeakForce QNM mode using silicon probes (RTESPA 300) with an 8 nm tip radius, 40 N m^–1^ spring constant and resonant frequency 300 kHz, or silicon nitride probes (ScanAyst air) with a 2 nm tip radius and 0.4 N m^–1^ spring constant. Scans (3 × 3 μm^2^) of at least three different locations were taken per sample. Images were analyzed by using the NanoScope Analysis 2.0 software. Topography images were corrected by subtracting a second-order polynomial background.

### Scanning Electron Microscopy (SEM) and Energy-Dispersive X-ray Spectroscopy (EDS)

Surface and cross-sectional images were acquired using a JEOL JSM-7100F field emission gun scanning electron microscope. Elemental analysis and EDS maps were acquired by using an Oxford Instruments X-Max 80 mm^2^ detector. Films were coated with gold and palladium to improve conductivity. Cross sections were prepared by fracturing the films in liquid nitrogen. An accelerating voltage of 5 keV was used for imaging to minimize sample damage, while a higher voltage of 20 keV was used for EDS measurements.

### Water Contact Angle Measurements (WCA)

WCA measurements of the coatings before and after antibacterial testing were conducted using a DSA100 KRUSS drop shape analyzer with the sessile drop method. A 10 μL deionized water droplet was deposited onto the sample using a micropipette, and 20 successive contact angle measurements were taken with a 1 s interval between them. The procedure was repeated at three different positions on each film, and the average value was calculated. All measurements were performed at room temperature (21 ± 1 °C).

### Numerical Simulations

In the same spirit as in ref (42), Brownian dynamics simulations were performed (implicit solvent technique) for a binary mixture composed of *N*_L_ latex and *N*_T_ TiO_2_ particles. The time evolution of the system was obtained by solving the corresponding coupled overdamped Langevin equations of motion.^45^ The system was initialized by randomly placing the particles in a box with *L*_*x*_ × *L*_*y*_ × *L*_*z*_ = 25σ_L_ × 25σ_L_ × 150σ_L_, where σ_L_ is the diameter of the latex particles, without overlap.^46^ Note that here we consider σ_L_ = 4σ_T_, where σ_T_ is the diameter of the TiO_2_ particles. Thermal fluctuations were obtained from a Gaussian distribution with standard deviation , where d*t* = 10^–6^τ is the Brownian time-step with time unit τ = σ_L_^2^/*D*_L_, and *D_i_* the bare diffusion coefficient of the particles of species *i* = L,T. The diffusion coefficients *D*_L_ and *D*_T_ were set to *D*_L_ = σ_L_^2^/τ and to *D*_T_ = *D*_L_σ_L_/σ_T_, respectively. The system was confined between two parallel reflective walls (along the *x* and *y*-axis, on which we use periodic boundary conditions) acting on the surface of the particles. The upper wall descends over time from the initial position *z*_0_ = 150σ_L_ to different values of *z*_f_ and at different speeds, depending on the studied case, described below. This process was used to model water evaporation.^47−49^ The steric interaction between latex particles was modeled via cut 12–6 Lennard-Jones potentials of the form
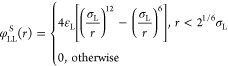
1where ε_L_ = *k*_B_*T* defines the interaction strength. Furthermore, colloidal stability of the latex particles was achieved via screened electrostatic interactions of the form
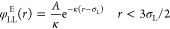
2where κ is the inverse screening length, here fixed at κ = 10σ_L_ throughout, and *A* = 30*k*_B_*T*σ_L_^–1^ sets the interaction strength per unit length. On the other hand, the interaction between TiO_2_ particles was modeled by the combination of an 18–9 LJ potential and screened electrostatic repulsion, namely
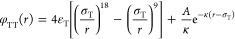
3where ε_T_ sets the attraction strength, here fixed at 4*k*_B_*T*. The first term was truncated at 2σ_T_, whereas the second was truncated at 7σ_T_/2. This combination, phenomenologically similar to the well-known DLVO potential,^50^ allows the TiO_2_ particles to aggregate weakly. Finally, the cross-species interaction was modeled via a steric and an electrostatic contribution. The first is equivalent to eq 1 yet replacing σ_L_ with σ_LT_ = (σ_L_ + σ_T_)/2, the average diameter of the two particle types. On the other hand, the electrostatic interactions were defined as
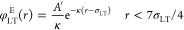
4

The value of *A*′ varied depending on the studied case, as discussed below. All of the interactions described above were shifted to zero at the cutoff distance, in terms of both potentials and forces. All simulations were run with the LAMMPS^51^ 23 June 2022 stable version with optimized neighbor lists.^52,53^

#### Different Cases

In this work, two evaporation rates were studied. In the fast evaporation case, we run 10 × 10^6^ time-steps, corresponding to 10τ, whereas in the slow evaporation case, we run 400 × 10^6^ time-steps, corresponding to 400τ. We also studied two different concentrations. In the low-concentration case, *N*_L_ = 2000 and *N*_T_ = 38,000,38000, corresponding to an initial occupied volume fraction of ≈1.4%, whereas in the high-concentration case, *N*_L_ = 2000 and *N*_T_ = 98000, corresponding to an initial occupied volume fraction of ≈2.0%. In the former case, the final position of the upper wall was *z*_f_ = 5.15σ_L_, whereas in the latter *z*_f_ = 7σ_L_. This corresponds, in both cases, to a final occupied volume fraction of ≈42%. Furthermore, the interactions between latex and TiO_2_ particles can be either attractive or repulsive. In the case in which latex and TiO_2_ have the same charge, *A*′ = 30*k*_B_*T*σ_L_^–1^, whereas in the case in which the charges differ, *A*′ = −30*k*_B_*T*σ_L_^–1^.

### Antibacterial Testing

*S. aureus* (ATCC 43300, methicillin-resistant, MRSA) was maintained on tryptone soy agar (TSA, Oxoid), and a single colony was used to inoculate tryptone soy broth cultures when required. Liquid cultures (10 mL) were set up in sterile 50 mL centrifuge tubes (Thermo Fisher) and incubated at 37 °C while shaking at 150 rpm overnight for 20 h. These overnight cultures routinely contained 1 × 10^9^ colony-forming units per mL, and standard bacterial suspensions for testing of approximately 1 × 10^6^ colony-forming units (CFU) per mL were obtained after serial dilutions in 1/500 nutrient broth (CM0001, OXOID) and then stored in the refrigerator at 5 °C. Photocatalytic activity of the 18 mm × 18 mm coatings was evaluated by the adhesive film test based on ISO 27447:2009.^54^ The coatings were soaked for 15 min in 70% IPA prior to the test for disinfection purposes and then dried at 37 °C. Then, they were placed in a Petri dish on square metal plates (previously soaked in 70% IPA) with wet filter paper underneath to keep a moist environment. A 100 μL aliquot of the standard MRSA suspension (approximately 10^5^ CFU) was applied to each coating sample and polypropylene films (15 mm × 15 mm pieces, sterilized by UV treatment for 15 min) were placed on top of the bacterial suspension, as per ISO 27447 requirements to keep the bacterial suspension from drying out. A piece of borosilicate glass (10 cm × 10 cm) was placed on top of each Petri dish for further moisture conservation. The coatings were irradiated with a 368 nm, 15 W Black Light Blue (BLB) bulb (Sankyo Denki) with an irradiance of 0.249 mW cm^–2^ (bulb placed at a 46.5 cm distance from the coatings) for 4 h. After irradiation, the coatings were immersed in 10 mL of Tryptone soya broth (Oxoid) with 7 g Tween 80 (Sigma-Aldrich) and 1 g Lecithin (Thermofisher) per liter (SCDLP broth), and vortexed for 10 s at 2500 rpm to recover bacteria. The recovery solution was then serially diluted (10^–1^–10^–4^) in phosphate-buffered saline solution, and a 1 mL aliquot was used to prepare pour plates of TSA. Once these were set, they were incubated at 37 °C for 24 h prior to colony counting to determine the number of viable cells. Control tests on uncoated glass coverslips, as well as tests of the coated samples and glass coverslips in the dark were also carried out following the same procedures. Tests were performed on three replicates of each coating/control, and data were expressed as the mean and standard deviation of recovered viable bacteria.

## Results and Discussion

**Figure 1:**
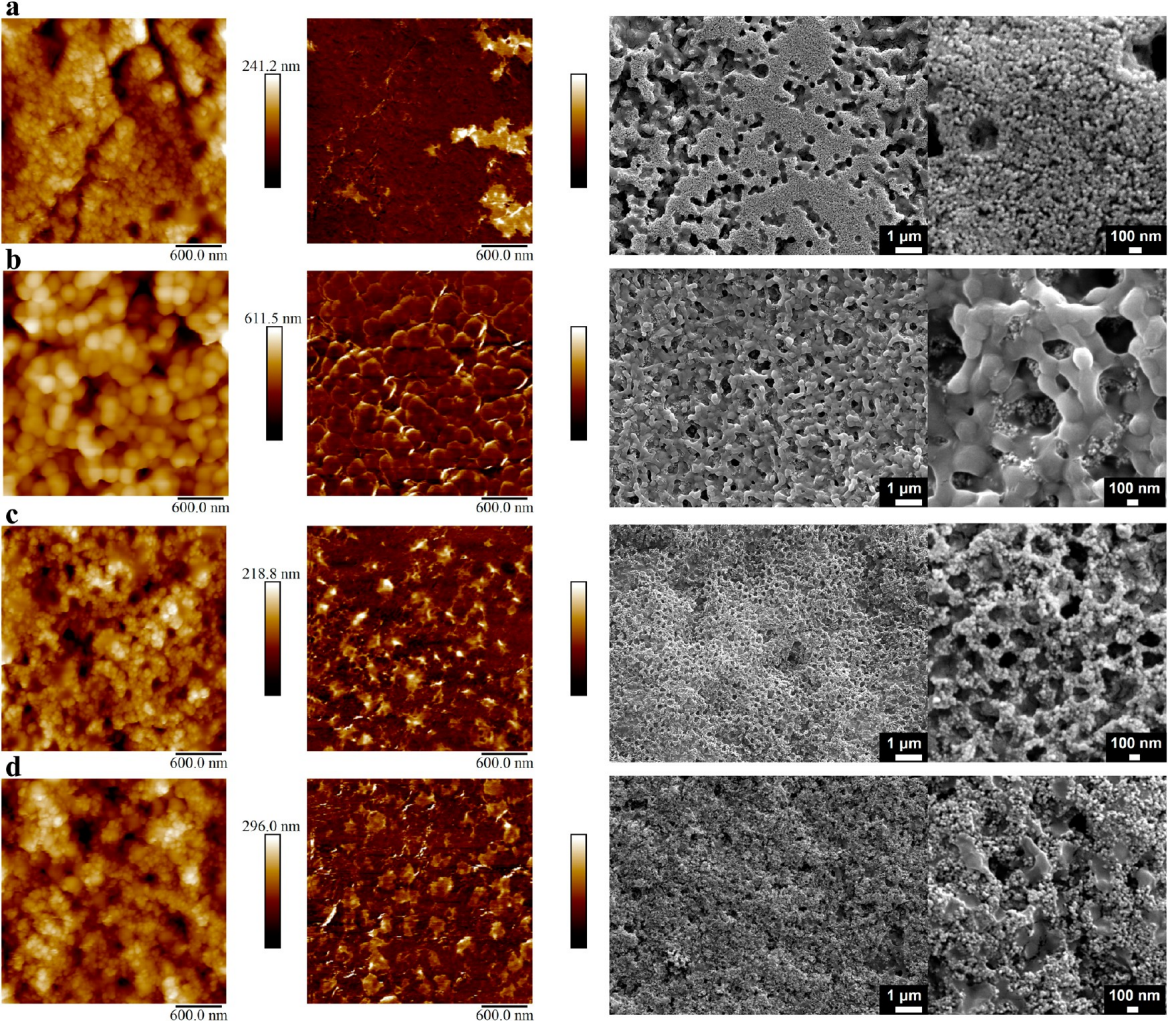
3 ×3 μm^2^ AFM topography images (left column), corresponding adhesion maps (in arbitrary units, order of 1 nN) (middle), and SEM images (right column) of the top surface of films formed from samples L + Ti_30,fast_ (a), L + Ti_30,slow_ (b), L – Ti_30,fast_ (c), and L – Ti_30,slow_ (d).

**Figure 2:**
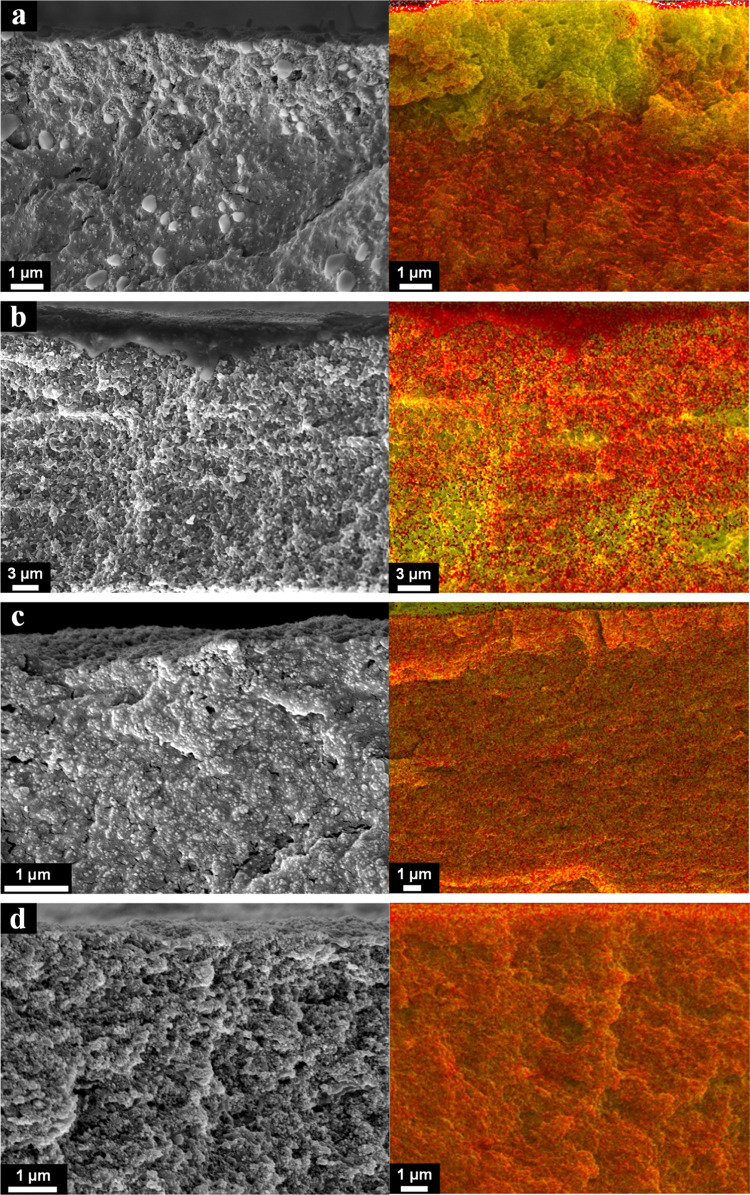
(Left) SEM cross-sectional images of films formed from blends (a) L + Ti_30,fast_, (b) L + Ti_30,slow_, (c) L – Ti_30,fast_, and (d) L – Ti_30,slow_. (Right) Cross-sectional EDS maps, showing titanium and carbon in yellow and red colors, respectively. Nonmerged SEM-EDS images are shown in Figure S2.

To investigate the impact of particle interactions and evaporation rate on the film microstructure and photocatalytic properties, we prepared colloidal blends containing TiO_2_ and either positively or negatively charged polymer particles, referred to as latex particles from now onward, and film formed them under different environmental conditions (see Table 1). The average hydrodynamic diameters (*D*_H_) for TiO_2_, positively, and negatively charged latex particles were 45 ± 1, 297 ± 2, and 211 ± 1 nm, respectively (Figure S1). ζ-Potentials of TiO_2_, positive, and negative latex particles were +40 ± 1, +39 ± 1, and −46 ± 1 mV, respectively, as determined by electrophoretic light scattering. Atomic force microscopy (AFM) and scanning electron microscopy (SEM) were used to image the top surface of the dried films. Figure 1 shows topography and adhesion maps for TiO_2_/positive latex or TiO_2_/negative latex films formed at fast (60 ± 1 °C, 10 ± 5% RH), or slow (21 ± 1 °C and 50 ± 5% RH) drying conditions and SEM images of the film top surface. Figure 2 shows the corresponding SEM cross-sectional images and EDS maps (nonmerged SEM-EDS images are shown in Figure S2).

**Table 1:** TiO_2_/Latex Blend Formulations and Film Formation Conditions

sample name	TiO_2_ ζ^a^ [mV]	latex ζ^a^ [mV]	TiO_2_/latex weight ratio^b^	Pe_S_^c^	*T*^d^ [°C]	RH^e^ [%]
L + Ti_30,fast_	40 ± 1	39 ± 1	30:70	25.6	60 ± 1	10 ± 5
L + Ti_30,slow_	40 ± 1	39 ± 1	30:70	14.2	21 ± 1	50 ± 5
L – Ti_30,fast_	40 ± 1	–46 ± 1	30:70	25.6	60 ± 1	10 ± 5
L – Ti_30,slow_	40 ± 1	–46 ± 1	30:70	14.2	21 ± 1	50 ± 5
L + Ti_50,fast_	40 ± 1	39 ± 1	50:50	25.6	60 ± 1	10 ± 5

a ζ-Potential as measured by ELS.

b The weight ratio is based on the mass of the polymer and TiO_2_ particles excluding the mass of water.

c Péclet number of small particles (see the Supporting Information for calculations).

d Film formation temperature.

e Relative humidity.

Starting with the TiO_2_/positive latex film (see Figure S3a) formed at fast evaporation conditions (L + Ti_30,fast_), AFM reveals a surface predominantly occupied by small TiO_2_ particles (Figure 1a, left). This observation is confirmed by the corresponding SEM images (Figure 1a, right). The surface adhesion map (Figure 1a, middle) reveals regions covered by small TiO_2_ particles, appearing in a darker color due to their reduced adhesion. This enables the detection of cavities located between the TiO_2_ domains. These have similar size and shape when compared to the latex particles. This indicates that the arrangement of TiO_2_ particles is templated by a bed of packed latex particles, similarly to what was reported in silica/latex binary films.^35^ Indeed, the SEM cross section reveals a TiO_2_-enriched layer at the film’s surface (Figure 2a, left) with a layer of mainly latex particles underneath. The EDS map (Figure 2a, right) confirms the stratified small-on-top architecture of the film, depicted by the top of the film enriched in Ti (in yellow) and the rest enriched in polymer (carbon is shown in red).

For TiO_2_/positive latex films dried at slow evaporation conditions (L + Ti_30,slow_), AFM topography and adhesion maps (Figure 1b, left and middle) show latex particles covering the top surface. A closer examination of the SEM images (Figure 1b, right) reveals latex particles on the top surface, with TiO_2_ particles underneath or filling gaps between them. In the cross-sectional SEM images and EDS maps (Figure 2b) a coalesced, thin layer, possibly a monolayer, of latex particles can be observed at the top. While simulations have previously indicated the presence of a monolayer of large particles at the top surface,^40,41^ experimental evidence for this phenomenon has rarely been observed.^39^ The EDS map confirms the presence of a carbon-rich layer at the top of the coating (in red). Underneath that layer, the distribution of small and large particles is nonuniform, with distinct regions exhibiting an enrichment of either latex or TiO_2_ particles.

The microstructures observed experimentally for L + Ti_30,fast_ and L + Ti_30,slow_ were compared to those predicted by the ZJD^55^ and Schulz-Sear models,^56^ as these two systems are composed of like-charged particles. The ZJD model accurately predicts small-on-top stratification for L + Ti_30,fast_, and L + Ti_30,slow_ as borderline conditions, which overall results in good agreement. Notably, the Sear model predicts that neither of the two systems will stratify (Figures S4 and S5). The latter prediction is in contrast with our experimental observations for the L + Ti_30,fast_ system, which exhibited clear small-on-top stratification. In our work, we use nonspherical TiO_2_ particles with a high Hamaker constant. It is known that these factors, together with hydrodynamic interactions can lead to deviation of predictions from experimental results.^33^

We now move on to the TiO_2_/negative latex film dried at fast evaporation conditions (L – Ti_30,fast_). AFM topography and adhesion maps, together with top-view SEM images (Figure 1c), provide evidence for a large number of titania particles forming a honeycomb structure at the surface of the film. However, the SEM cross section (Figure 2c, left) illustrates a uniform film with small and large particles distributed throughout the entire depth of the film. This is confirmed by the EDS map (Figure 2c, right), depicting the film orange, indicating the coexistence of Ti (in yellow) and C (in red). The interaction between TiO_2_ and latex involves collisions between them and may lead to the eventual adsorption of small particles to the surface of large particles, followed by their collective diffusion within the aqueous medium. This hypothesis is supported by our DLS experiments, which showed evidence of adsorption of TiO_2_ nanoparticles on latex particles in the dispersion (Figure S3b,c), possibly due to electrostatic interactions. To fully cover the surface of a latex particle, we would need ∼80 TiO_2_ particles.^57^ In our experiments, the number ratio of TiO_2_ to latex is ∼13, indicating that there would theoretically be no free TiO_2_ in the system if all of it was attached to the latex particles. Upon blending TiO_2_ with latex, the dispersion was vortexed for a few seconds and then immediately cast onto a substrate. While electrostatic attraction can lead to the formation of raspberry-like particles, a longer equilibration time might be necessary for all TiO_2_ particles to fully attach to the latex. A study by Eren et al.^58^ demonstrated the attachment of small, positively charged silica nanoparticles onto larger polystyrene latex nanoparticles, creating a raspberry-like structure. This process was found to be time-dependent, requiring interaction, attachment, and stabilization. Indeed, not all silica nanoparticles were immediately incorporated into the raspberry structures. This resulted in the presence of free silica nanoparticles in the solution, alongside raspberry particles. The presence of free TiO_2_ in our dispersions could explain the observed thin layer of small particles on top of the film (see Figure 1c). Due to their smaller size, TiO_2_ particles have larger mobility compared to the latex particles. Under fast evaporation conditions, free TiO_2_ particles may get trapped at the air–water interface before they attach to the latex surface. Underneath, a homogeneous distribution of raspberry-like particles can be found, contributing to the observed microstructure of the dried film.

When the same TiO_2_/negative latex formulation was subjected to slow evaporation conditions (L – Ti_30,slow_), the AFM topography and adhesion maps (Figure 1d, left and middle) of the resulting films showed no significant differences. SEM images of the top surface (Figure 1d, right) and cross section of the film (Figure 2d, left) evidence a homogeneous distribution of small and large particles. This is confirmed by the orange color in the EDS map (Figure 2d, right). Therefore, at a slower evaporation rate, the thin TiO_2_ layer at the top film surface is absent. With slower drying, the water–air interface is not descending fast enough to trap small particles and provides extended time and thus an increased number of collisions between TiO_2_ and latex particles to form raspberry particles.

**Figure 3:**
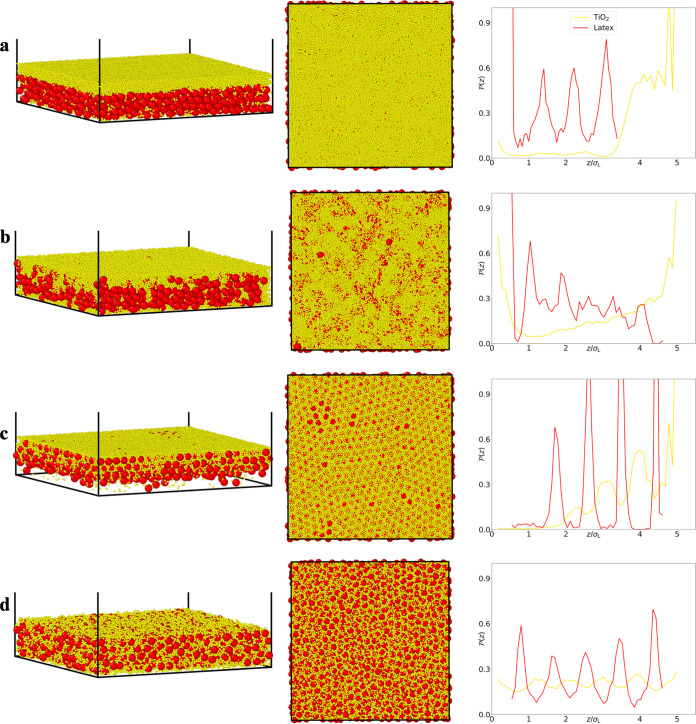
Brownian dynamics simulations results. Snapshots of a side view (left) and the top surface (middle) of the films taken at the end of the drying process. Particle probability distributions along the direction perpendicular to the film surface (right) for systems with repulsive interparticle interactions dried at (a) fast and (b) slow evaporation conditions, or attractive interparticle interactions dried at (c) fast or (d) slow evaporation conditions. Small (TiO_2_) and large (latex) particles are colored yellow and red, respectively.

Figure 3 shows the Brownian dynamics simulations that were carried out to model our experimental systems. When the interaction between small (TiO_2_) and large (latex) particles is repulsive and the evaporation rate is fast (Figure 3a), a distinct and uniform layer of small particles forms at the top of the film. This observation is corroborated by the corresponding particle probability distribution plots, indicating a clear enhancement of the probability of finding TiO_2_ particles at the top surface. Keeping the same repulsive interactions between small and large particles, but slowing down the evaporation rate (Figure 3b), results in a notably heightened presence of TiO_2_ particles at both the bottom and top surfaces in the dried film. The latter observation is likely to be linked to a weak aggregation of small particles which results in an increase in effective particle size and therefore segregation to the bottom surface. When interactions between small and large particles switch to attractive, at a fast evaporation rate (Figure 3c) simulations predict the alternating presence of latex and TiO_2_ particles throughout the dried film thickness, with an enhanced presence of small particles at the top surface. When slowing down the evaporation rate (Figure 3d) a more homogeneous distribution of large and small particles is observed. Overall, the simulations reproduce the microstructures we encountered experimentally and therefore support our hypothesis for the processes and phenomena involved in the assembly process.

**Figure 4:**
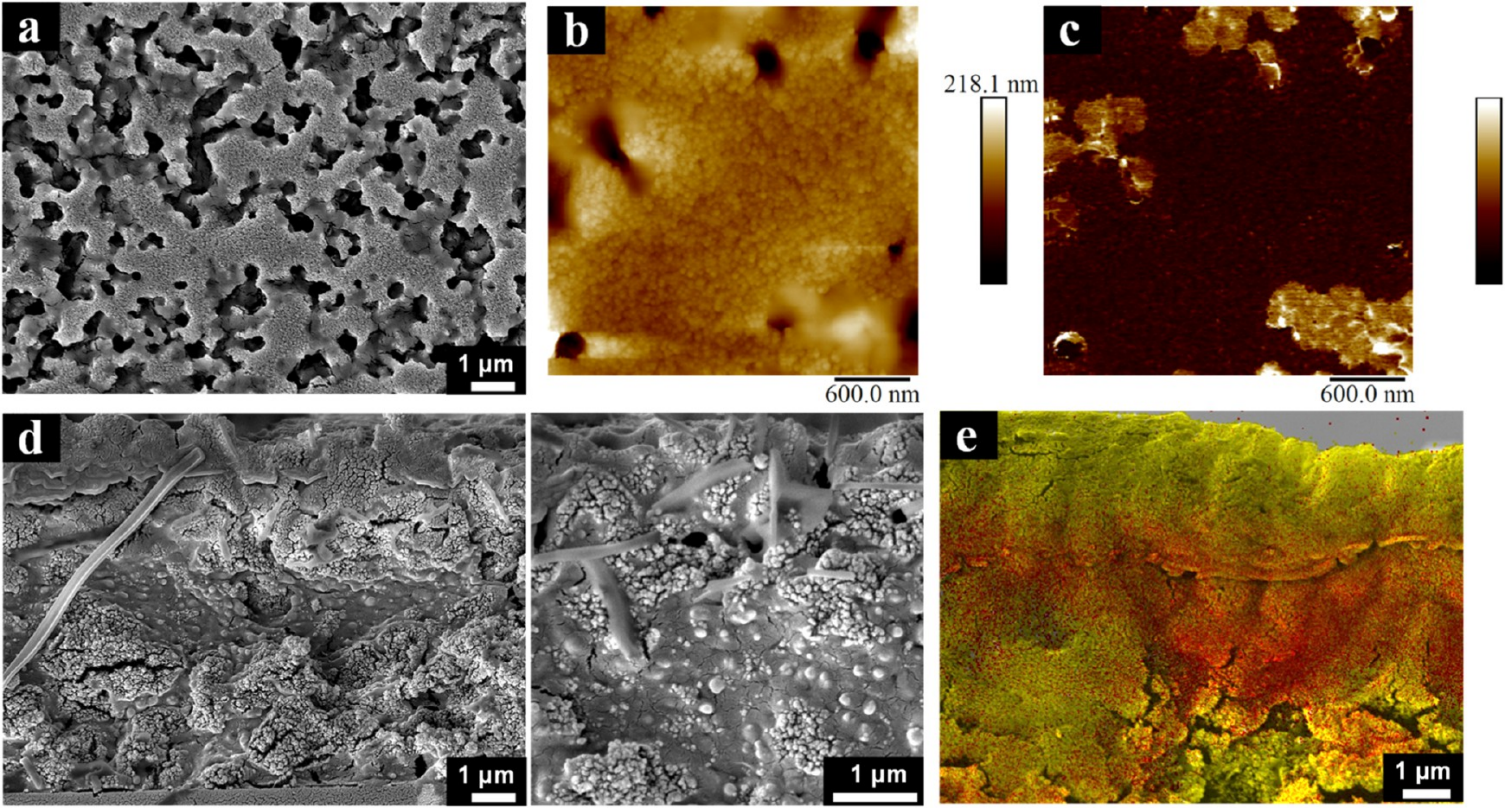
Characterization of the L + Ti_50,fast_ film. (a) SEM micrograph, (b) AFM topography, and (c) adhesion map (adhesion in arbitrary units, order of 1 nN) of the top film surface. (d) SEM cross-sectional images and (e) the corresponding cross-sectional EDS map, showing titanium and carbon in yellow and red, respectively.

After examining the microstructures of our films, it would be logical to predict that L + Ti_30,fast_ would perform best as a photocatalytic antibacterial coating. The small-on-top stratification in this film, concentrating TiO_2_ at the surface, should ensure maximum contact with bacteria as well as TiO_2_ accessibility to light for the formation of ROS. A fifth coating was developed to enhance further this favorable microstructure, namely, L + Ti_50,fast_. In this sample, the TiO_2_/latex weight ratio was increased to 50:50 (see Table 1). This approach aimed to augment the thickness of the top TiO_2_ layer to enhance the photocatalytic activity of the film. The top surface of the resulting L + Ti_50,fast_ film is indeed covered by small TiO_2_ particles, as demonstrated by SEM imaging (Figure 4a), AFM topography, and adhesion maps (Figure 4b,c). However, when the cross section of the film was examined, both a bottom and top layer of TiO_2_ were observed, with an intermediate latex-rich layer (Figure 4d,e). This architecture is similar to the sandwich structures observed in polystyrene/silica colloidal films using small-angle X-ray scattering (SAXS).^38^ The mechanism underlying the formation of such structures has not yet been explored, as theoretical studies have primarily concentrated on small-on-top or large-on-top stratification. We believe this structure has its origin on the agglomeration tendency of TiO_2_. TiO_2_ nanoparticles present a ζ-potential of 40 mV, which is an indicator for strong colloidal stability.^59^ However, van der Waals forces between TiO_2_ particles are stronger than those between latex particles, as a result of a larger contrast in dielectric constant with water and therefore a larger Hamaker constant value.^60^ Moreover, with the volume fraction of TiO_2_ doubled for L + Ti_50,fast_ compared to L + Ti_30,fast_, the number of particle collisions is significantly increased. This results in a higher probability of TiO_2_ aggregate formation. These aggregates might then diffuse slower than the free particles, which can possibly undergo size segregation and be accumulated above them.^33^ Brownian dynamics simulations (Figure S6) do predict the thicker (compared to L + Ti_30,fast_) and uniform top TiO_2_ layer for this system. However, the simulations can only partially capture a small increased tendency of the TiO_2_ particles to segregate at the bottom layer of the overall sandwich structure. The simulations incorporated weak aggregation between TiO_2_ particles to account for the attractive component of the DLVO-like potential. It is plausible that the expected weak attraction between TiO_2_ particles is not the sole factor responsible for the formation of the bottom layer, suggesting a more intricate mechanism that requires further investigation.

One of the aims of our study was to establish a connection between the microstructure and the photocatalytic and antibacterial properties of the film. The viability of MRSA, a prevalent source of hospital infections,^61^ on our films was assessed using the ISO 27447 protocol for photocatalytic surfaces.^54^ After an initial disinfection of the films with isopropyl alcohol (IPA) we evaluated the robustness of the films before testing by immersing them for 24 h in water and for 15 min in 70% IPA. Optical microscopy images of the films before and after soaking in 70% IPA can be seen in Figure S7, while AFM topography maps of the film top surface after soaking in water for 24 h or IPA for 15 min are shown in Figure S8. Among the 5 systems tested, only L + Ti_30,slow_ exhibited pronounced structural changes. Furthermore, macroscopic observation reveals that this film undergoes delamination after soaking in IPA (Figure S7b), and as a result, it was not subjected to further testing.

**Figure 5:**
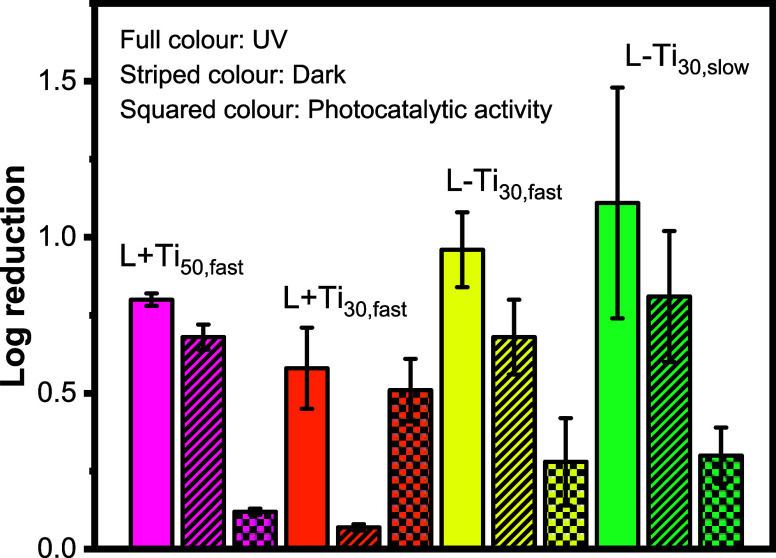
Reduction in the number of MRSA colonies on coating systems under UVA irradiation for 4 h or in the dark for 4 h. The numbers displayed represent the mean from triplicate samples. Photocatalytic activity is obtained after subtracting the activity in the dark from the activity under UV (log reduction UV – log reduction Dark). The number of surviving bacteria on each film was normalized to the control (number of surviving bacterial colonies on a bare glass substrate). This graph refers to the second (−2) logarithmic dilution of the initial recovery solution. Refer to Table S1 for the raw data.

MRSA was inoculated on the four remaining coating systems and on control glass substrates, and then, it was either irradiated with a UVA lamp or left in the dark for 4 h (control conditions). The MRSA reduction results after inoculation are presented in Figure 5. The number of surviving bacteria after testing was normalized to those of the control glass substrates. A minor reduction in bacterial counts was observed on the control samples following UV irradiation, resulting in a log reduction of 0.02–0.17 (see Table S1), which was subtracted from the corresponding MRSA reduction on the coated samples. Photocatalytic activity is defined as the activity under UV light after subtracting the activity in the dark. The raw data for MRSA reduction is available in the Supporting Information (SI) in Table S1. The logarithmic reduction in the number of viable MRSA cells after UVA irradiation for L + Ti_50,fast_, L + Ti_30,fast_, L – Ti_30,fast_, and L – Ti_30,slow_ is 0.80, 0.58, 0.69, and 1.11, while in the dark, it is 0.68, 0.07, 0.68, and 0.81, respectively, and the calculated photocatalytic activity is 0.12, 0.51, 0.26, and 0.30, respectively.

The first observation is that all of our coatings present photocatalytic activity to various degrees and that this results in an effective reduction of MRSA viability on them. Moreover, increasing the amount of TiO_2_ in the formulation led to an increase in the bactericidal activity of the L + Ti_50,fast_ system with respect to that of L + Ti_30,fast_. Surprisingly, neither of these two systems with predominantly TiO_2_ nanoparticles at the top are the best performing when it comes to reducing MRSA viability. Indeed, the antibacterial testing proved that the L – Ti_30,fast_ and L – Ti_30,slow_ systems present stronger antibacterial activity. We believe that this arises from the pores and cracks in these films, which provide additional accessibility for light and water to penetrate. This would, in turn, increase the amount and mobility of reactive oxygen species that are generated from TiO_2_ photocatalysis.

Another observation is that the reduction in MRSA viability is, overall, relatively low. TiO_2_ requires efficient radiation absorption when in contact with water and oxygen to produce ROS. Numerous studies mention that weak UV illumination is sufficient for TiO_2_ photocatalysis,^62−64^ but for Aeroxide TiO_2_ P25, this activity is much stronger in the UVB range compared to UVA.^65,66^ Indeed, the absorption spectrum of our TiO_2_ nanoparticle dispersion (Figure S9) shows that it primarily absorbs in the UVB (280–320 nm) and UVC (200–280 nm) region. This could explain the low photocatalytic activity of the films. We adhered to the ISO 27447 protocol for our testing, which requires UVA illumination, highlighting the need for new testing protocols that take absorption spectra aspects into consideration.

Last but not least, it is worth noting that most of the systems had antibacterial activity in the dark. We are not the first study to report on the biocidal effect of TiO_2_ nanomaterials in the absence of light.^67^ In an early study, Saito et al.^68^ showed evidence of cell wall disruption of *Streptococcus sobrinus* by coaggregates of TiO_2_ P25 nanoparticles post-illumination.^67^ More recently, Kiwi et al. showed TEM images of dead *Escherichia coli* after contact with TiO_2_-polyester films in the dark without any prior light treatment.^69^ This observation was explained by the neutral charge of nanoparticles close to the isoelectric point, which induces aggregation of TiO_2_ and possibly van der Waals attraction to the cell wall. To investigate whether this scenario could be accountable for the bactericidal activity of our films in the dark, L + Ti_50,fast_, L + Ti_30,fast_, L – Ti_30,fast_, and L – Ti_30,slow_ were examined for any signs of TiO_2_ agglomerate release. 100 μL of water was deposited on each of the four intact films and then retrieved after 4 h. After dilution in a total volume of 1 mL for subsequent DLS analysis (Figure S10), objects of around 214–312 nm were detected in the solution. Interestingly, this was observed only for three samples, L + Ti_50,fast_, L – Ti_30,fast_, and L – Ti_30,slow_, the same ones that exhibited activity in the dark. These agglomerates, formed by TiO_2_ nanoparticles and, less likely, latex particles, could interact with and damage MRSA cell walls.

After antibacterial testing, the films were resoaked in IPA for 15 min for disinfection purposes and characterized by AFM. Figure S11 reveals that the film surface shows increased presence of latex particles after two cycles of IPA cleaning and bacterial inoculation under UV light for 4 h. This indicates that overall the coatings are fairly robust, especially considering that the ISO 27447:2009 protocol requires prolonged soaking, which is not the situation for coatings in actual healthcare environments. UV exposure can, in some cases, lead to the degradation of organic binders within the coating. This degradation is caused by ROS generated during the photocatalysis of TiO_2_. As the duration of UV irradiation increases, pores begin to form on the coating surface. These pores gradually expose more TiO_2_ particles, further promoting photocatalysis.^30^ This phenomenon might contribute to the increased porosity and cracks observed in the L – Ti_30,fast_ and L – Ti_30,slow_ coatings after UV exposure (Figure S12). Interestingly, this effect was not observed in the L + Ti_50,fast_, L + Ti_30,fast_ films. Here, the presence of a thick top layer of TiO_2_ could mitigate binder degradation by protecting the underlying organic material. Additionally, yellowing of the coating after UV treatment has been linked to binder photodegradation.^30^ We did not observe any color change in our TiO_2_/latex coatings, potentially indicating reduced binder degradation.

Supplementary water contact angle (WCA) measurements for the four films before and after UV exposure and antibacterial testing are listed in Table S2. Films with a top TiO_2_ layer exhibited WCAs between 73 and 79°, consistent with values reported in the literature for weakly hydrophilic TiO_2_ coatings.^30,70^ Notably, the WCA remained unchanged (within experimental error) after the photocatalytic experiment, suggesting that the top TiO_2_ layer protected the underlying coating and mitigated significant surface alterations during testing. In contrast, the raspberry particle coatings displayed larger WCA values, indicating enhanced hydrophobicity. Interestingly, these values slightly increased after testing. While one might expect more defects in the coating (cracks and porosity) to lead to higher wettability due to increased surface area and water contact points, such features in thin films can trap air, leading to increased hydrophobicity and higher WCA.^71−73^ A more hydrophobic surface, characterized by a higher contact angle, exhibits a lower wettability. This creates a less hospitable environment for bacteria, which require a certain level of hydration to survive and colonize.^74^ Surfaces with increased hydrophobicity have been proven to reduce bacterial adhesion^34^ and have enhanced photocatalytic activity^75^ compared to less hydrophobic surfaces. However, the increased hydrophobicity observed in the raspberry particle coatings did not result in a significant increase in antibacterial activity in the dark. This observation, together with the increased antibacterial efficiency under UV light compared to under dark conditions, strongly suggests that ROS generation plays a key role in the biocidal mechanism. Importantly, cracks and pores in the film increase water accessibility and can potentially expose more TiO_2_ to UV and oxygen, facilitating enhanced ROS production and transport.

## Conclusions

We harnessed size segregation and interparticle interactions in drying colloidal blends of large latex and small TiO_2_ nanoparticles to fabricate a portfolio of coatings with different microstructures. We achieved this by varying the temperature and relative humidity—and the particle surface charge, using either positively or negatively charged latex particles together with positively charged TiO_2_ nanoparticles. Five distinct film microstructures were obtained and characterized using AFM, SEM, and EDS. These include small-on-top stratification, a thin layer of large or small particles on top, homogeneous films of raspberry-type particles, and sandwich structures. Brownian dynamics simulations successfully modeled the majority of experimental results, although they fell short in replicating the latex monolayer in the L + Ti_30,slow_ system and the sandwich structure observed for the L + Ti_50,fast_ system. Mechanisms governing the formation of the latter structures are still elusive and there is a clear gap of understanding that needs to be filled, as very recent experimental and modeling work has shown.^76^

To correlate the photocatalytic and antibacterial activities of the coatings with their microstructure, we evaluated the viability of MRSA on our systems following the ISO 27447 standard method. Films with a TiO_2_-enriched top surface were robust and withstood soaking tests in water, IPA, and inoculation with bacteria solutions over prolonged periods of time. All of our coatings presented photocatalytic activity to various degrees, resulting in a 0.6–1.1 log reduction of MRSA. This, together with WCA results, confirms that a difference in surface hydrophilicity did not significantly impact antibacterial activity in the dark and indicates that the main mechanism of biocidal action is via ROS generation. The relatively low activity was attributed to the UVA irradiation required by the ISO standard, which falls outside of the best absorption range of the TiO_2_ nanoparticles. Most systems had antibacterial activity in the dark as well, and we demonstrated how this is due to the release of aggregates that are likely to disrupt bacterial cell walls.

Our work provides design rules for photocatalytic coatings based on the relationship between their performance and microstructure as well as demonstrates the power of evaporation-induced size segregation and interparticle interactions to control them. These rules are system-dependent, particularly regarding the particle charge and Peclet numbers. When using positively charged particles with a large-to-small size ratio of 6.6, faster evaporation conditions (Pe_S_ = 25.6) led to small-on-top stratification for a large-to-small weight ratio of 70:30. However, increasing the small particle content (weight ratio of 50:50) resulted in a sandwich structure and a bottom layer of small particles. Notably, coatings with a top TiO_2_ layer exhibited better stability upon soaking and antibacterial testing and a more homogeneous top surface. These findings suggest the existence of an optimal biocide concentration at which a small-on-top structure is retained, and the coating durability is maximized. Slower evaporation conditions (Pe_S_ = 14.2) resulted in a polymer layer on top and a film with reduced durability. Oppositely charged particles with a large-to-small size ratio of 4.6 formed raspberry particles in the formulation. These were homogeneously distributed within the film upon drying, with an enhanced TiO_2_ presence at the top surface when rapidly dried (Pe_S_ = 25.6). It is likely that cracks and increased porosity of such films are responsible for the enhanced photocatalytic activity in these systems, as they facilitate ROS production and transport.

Overall, our results suggest that particle charge, weight ratio, and Peclet number play crucial roles in the final structure and properties of the coating. Optimizing these factors can lead to coatings with the desired TiO_2_ distribution, photocatalytic activity, and durability. We believe that the insights presented here can be of high value not only to those developing antibacterial surfaces but also to those working on other applications of photocatalysis such as self-cleaning surfaces or water purification.

## Supporting Information Available

Synthesis of PDMAPMA and PSSNa macroRAFT agents; emulsion copolymerization of *n*-butyl acrylate and methyl methacrylate; intensity vs hydrodynamic diameter curves for TiO_2_ and latex nanoparticles; nonmerged SEM-EDS cross-sectional images; intensity vs hydrodynamic diameter curves and ζ-potential measurements for TiO_2_/latex blends; Peclet number calculations; change in mass over time for drying film at fast evaporation conditions; Pe_S_ and ϕ_s_ state diagram (Schulz-Sear/ZJD model); Brownian dynamics simulations to model the L + Ti_50,fast_ system; data spreadsheet for photocatalytic antibacterial testing of films; optical microscope images of films before and after cleaning with isopropyl alcohol; AFM topography images of films after soaking in water and isopropyl alcohol; UV–vis absorption spectrum of TiO_2_ dispersion; intensity vs hydrodynamic diameter curves from the aqueous solutions recovered from coatings after 4 h; AFM topography images of films after antibacterial testing and further disinfection; optical microscope images of films after antibacterial testing and further disinfection; and water contact angle measurements (PDF)
