# Supplementary material for: Tunable Assembly of Photocatalytic Colloidal Coatings for Antibacterial Applications
Source: ACS Appl Polym Mater. 2024 Aug 23;6(17):10298–310. doi: 10.1021/acsapm.4c01436 (PMC11406486; doi:10.1021/acsapm.4c01436)
Supplement: Supplementary file 1 — ap4c01436_si_001.pdf [file ap4c01436_si_001.pdf]

Supporting Information

Tunable assembly of photocatalytic colloidal coatings for antibacterial applications

Constantina Sofroniou[1], Alberto Scacchi[2], Huyen Le[1], Edgar Espinosa Rodriguez[3], Franck D'Agosto[3], Muriel Lansalot[3], Patrick S.M. Dunlop[4], Nigel G. Ternan[5], Ignacio Martín-Fabiani[1]*

1 Department of Materials, Loughborough University, Loughborough LE11 3TU, United Kingdom.

2 Department of Applied Physics, Aalto University, Aalto FI-00076, Finland; Department of Bioproducts and Biosystems, Aalto University, Aalto FI-00076, Finland; Department of Mechanical and Materials Engineering, University of Turku, Turku 20500, Finland.

3 Universite Claude Bernard Lyon 1, CPE Lyon, CNRS, UMR 5128, Catalysis, Polymerization, Processes and Materials (CP2M), Villeurbanne F-69616, France.

4 Nanotechnology and Integrated BioEngineering Centre (NIBEC), Ulster University, Newtownabbey BT37 0QB Northern Ireland, United Kingdom.

5 Nutrition Innovation Centre for Food and Health (NICHE), Ulster University, Londonderry BT52 1SA Northern Ireland, United Kingdom.

*corresponding author: Constantina Sofroniou, c.sofroniou@lboro.ac.uk

Synthesis of PDMAPMA and PSSNa macromolecular RAFT (macroRAFT) agents in water. The reactive macromolecular chain transfer agents (or macroRAFT) needed to prepare the latex particles were prepared by reversible addition-fragmentation chain transfer (RAFT) polymerization in solution.

PSSNa macroRAFT agent. The protocol used for the synthesis of PSSNa macroRAFT agent was adapted from the experimental conditions described by Velasquez et al.[1] First, 200 g of

deionised water were added to a 500 mL double-jacket glass reactor equipped with a condenser. CTPPA (1.7 g, 6.1 mmol), SSNa (19 g, 92 mmol) and sodium bicarbonate ($NaHCO_3$) (0.15 g, 1.8 mmol) were then added to water. The mixture was deoxygenated with nitrogen for 30 min and then heated to 80 °C, and the stirring was set at 250 rpm. To start the experiment, ACPA (0.15 g, 0.6 mmol) was added together with 1,3,5-trioxane (3.3 g, added as an internal reference to follow the kinetics via NMR) in 1 mL of deionised and deoxygenated water. Full conversion (determined by ^{1}H NMR) was achieved after 3 h. The recovered solution had a solids content of 8.5 %. A sample was dried and the polymer recovered was analysed by MALDI-ToF mass spectrometry to determine the molar mass of the macroRAFT agent: $M_{n(MALDI\text{-}TOF)}$ = 3021 g mol^{-1}.

PDMAPMA macroRAFT agent. The synthesis of the PDMAPMA macroRAFT was performed in water following the protocol described by Engström et al.[2] 200 g of deionised water were added to a 500 mL double-jacket glass reactor equipped with a condenser. CTPPA (3.6 g, 13 mmol) and DMAPMA (40 g, 235 mmol) were added to the reactor allowing to dissolve the RAFT agent and obtained a homogeneous solution. The addition of HCl was necessary to set the pH at 6. The mixture was then deoxygenated with nitrogen for 30 min and after that heated to 70 °C. The stirring was set at 250 rpm. AIBA (0.71 g, 2.6 mmol) was added together with 1,3,5-trioxane (3.6 g) in 1 mL of deionised and deoxygenated water to start the experiment. After 2 h of reaction, 96% conversion was achieved. The temperature of the reactor was decreased to 45 °C to recover the product. The polymer was precipitated three times with cold acetone, and finally dried in vacuum for 24 h at room temperature. The precipitated polymer was analysed by MALDI-ToF mass spectrometry to determine the molar mass of the macroRAFT agent: $M_{n(MALDI\text{-}TOF)}$ = 2850 g mol^{-1}.

Emulsion copolymerization of *n*-butyl acrylate and methyl methacrylate in the presence of PSSNa and PDMAPMA macroRAFT agents. Emulsion copolymerizations of BA and MMA (50/50 % in wt.) were performed at 70 °C in a 500 mL double-jacket glass reactor equipped with a condenser using either PSSNa or PDMAPMA macroRAFT agent (ensuring particle stabilization) and targeting a final solids content close to 20 wt%.

PSSNa-mediated emulsion copolymerization. 150 g of deionised water were poured into the reactor and deoxygenated for 30 min. BA (20 g, 0.15 mol) and MMA (20 g, 0.2 mol) were then added to the reactor, followed by 3.5 mL of the macroRAFT solution (0.35 g, 0.12 mmol). The mixture was stirred at 250 rpm and heated to 70 °C. 0.09 g of APS (0.39 mmol) dissolved in 1 mL of deionised and deoxygenated water was added to start the polymerization. The reaction was stopped after 4 h when full conversion was achieved, as determined by gravimetric analysis.

PDMAPMA-mediated emulsion copolymerization. 200 g of deionised water were poured into the reactor and deoxygenated for 30 min. BA (25 g, 0.195 mol) and MMA (25 g, 0.25 mol) were then added to the reactor, followed by 0.16 g of dry macroRAFT (0.053 mmol). The mixture was stirred at 250 rpm and heated to 70 °C. 0.11 g of APS (0.4 mmol) dissolved in 1 mL of deionised and deoxygenated water was added to start the polymerization. The reaction was stopped after 4 h when full conversion was achieved, as determined by gravimetric analysis.

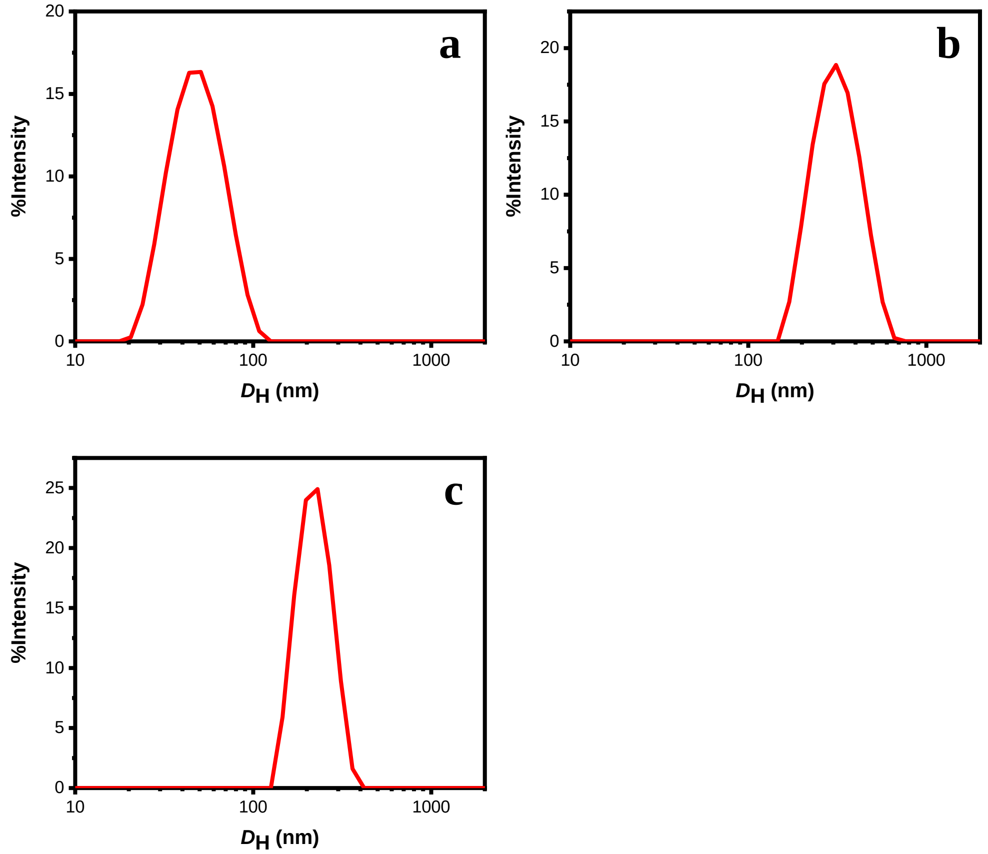

Figure S1. Intensity vs hydrodynamic diameter of (a) TiO_2 nanoparticles, (b) positively charged latex particles, and (c) negatively charged latex particles, measured by dynamic light scattering (DLS).

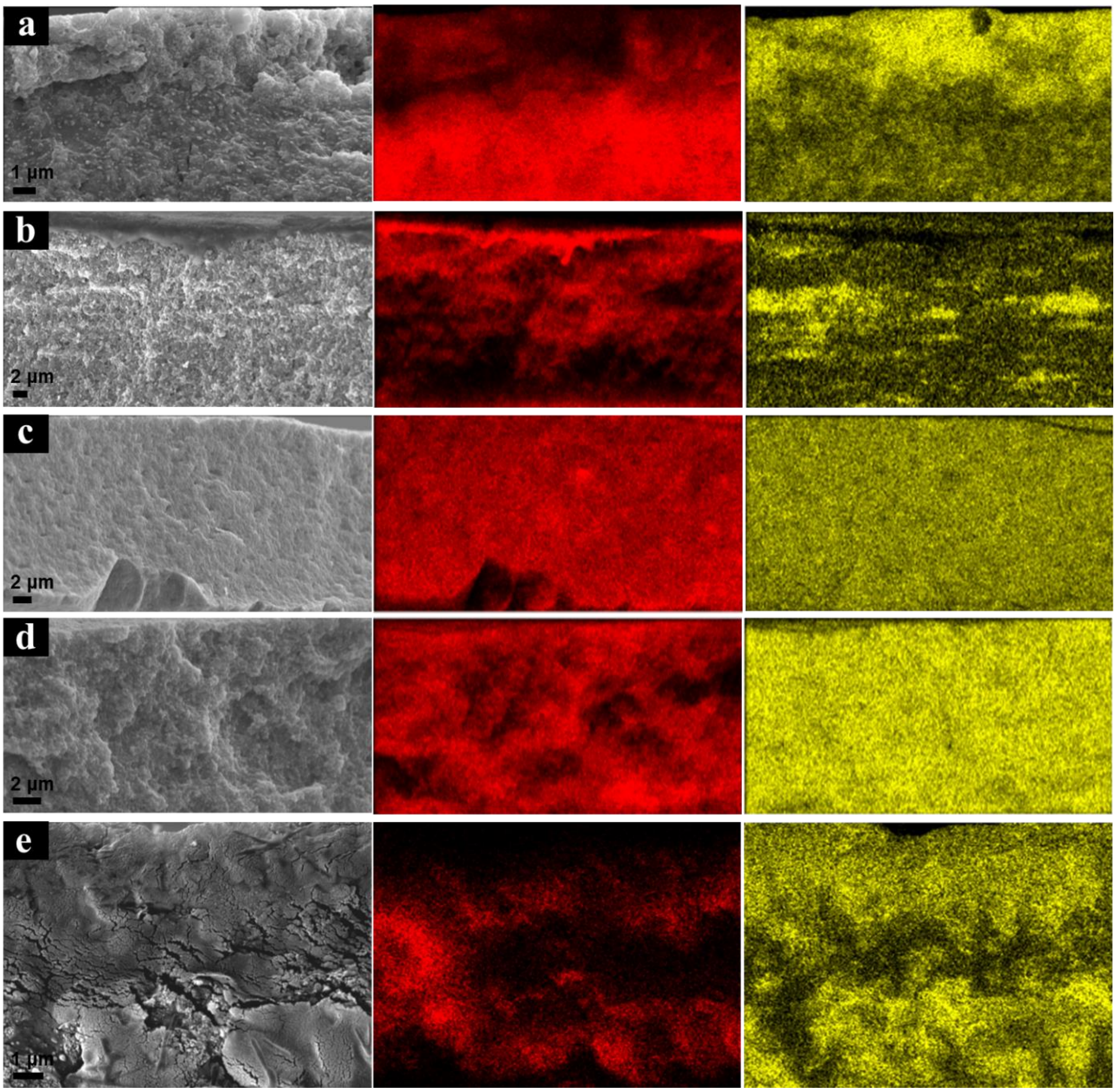

Figure S2. SEM cross-section images and EDS maps showing carbon and titanium in red and yellow colour, respectively, for the samples L+$Ti_{30,fast}$ (a), L+$Ti_{30,slow}$ (b), L-$Ti_{30,fast}$ (c), and L-$Ti_{30,slow}$ (d), L+$Ti_{50,fast}$ (e).

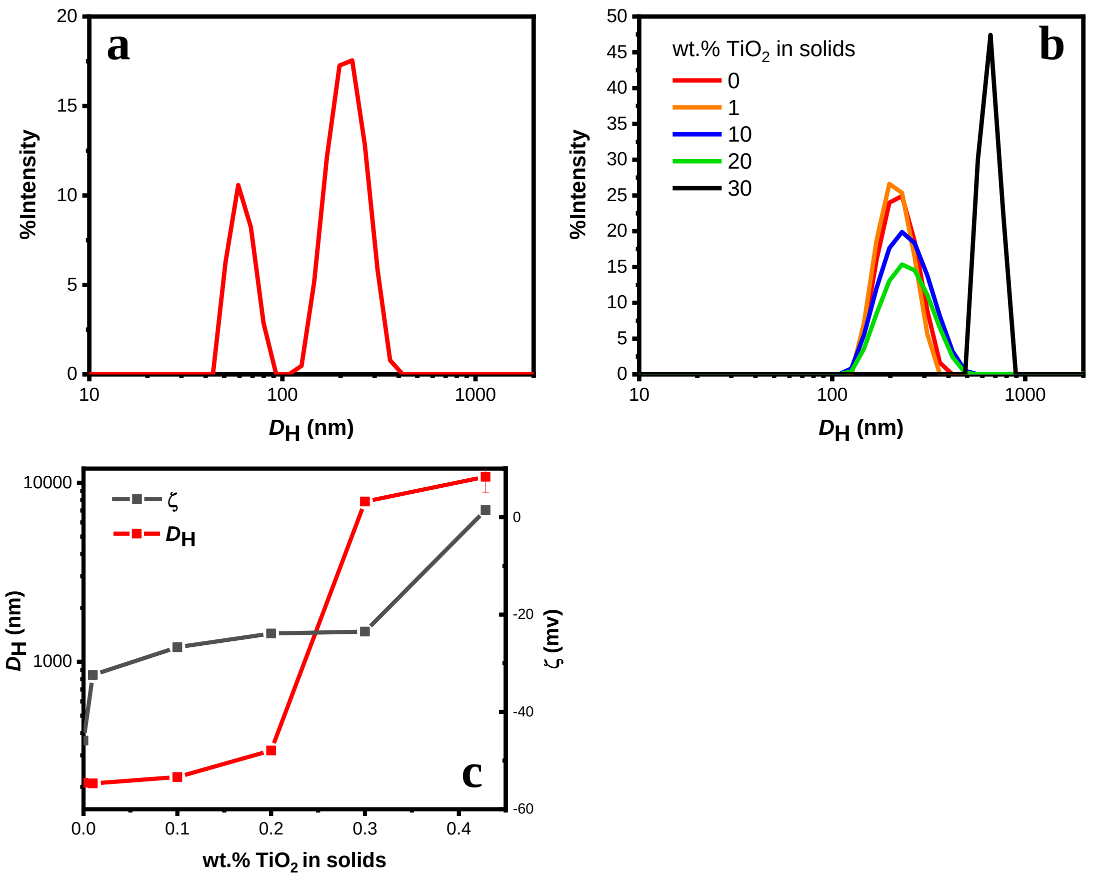

Figure S3. Intensity vs hydrodynamic diameter of (a) the TiO_2/positive latex blend, (b) the negative latex dispersion upon consecutive additions of TiO_2 nanoparticles, and (c) the evolution of hydrodynamic diameter and zeta potential (ζ) of the same blends.

Péclet Number Calculations

Péclet numbers for TiO_2 and positive latex particles have been calculated as follows:

$$P_e = \frac{6\pi\mu RH\dot{E}}{kT} \qquad Equation\ S1$$

Where μ is the viscosity of the solvent, R is the particle radius, H is the initial thickness of the wet film, $\dot{E}$ is the evaporation rate, k is the Boltzmann's constant, and T is the temperature in Kelvin. The particle radius was calculated as D_H / 2 from the values obtained from DLS (45 ± 1 nm and 297 ± 2 nm for TiO_2 and positively charged latex respectively). The initial film thickness was calculated to be 1.23 mm dividing the glass slide surface area (324 mm^2) by the casting volume (400 µl). The films were dried at slow and fast evaporation rates (see *Table 1*) and the value used for water viscosity was 1 × 10^{-3} (294 K) and 0.5 × 10^{-3} Pa.s (333 K) respectively. The value used for the slow evaporation rate was taken from Utgenannt *et al.*[3] (1.1 × 10^{-7} ms^{-1}). For the fast evaporation rate, the value was calculated to be 4.5 × 10^{-7} ms^{-1} using the change in film weight (arising from water evaporation) over time (Δ_m/Δ_t) while the film was drying in the oven (see *Figure S4*). The evaporation rate can be calculated as:

$$\dot{E} = \frac{\left(\frac{\Delta_m}{\Delta_t}\right)}{A\rho} \qquad Equation\ S2$$

Where A is the film surface area and ρ is the density of water (1.0 g cm^{-3}).

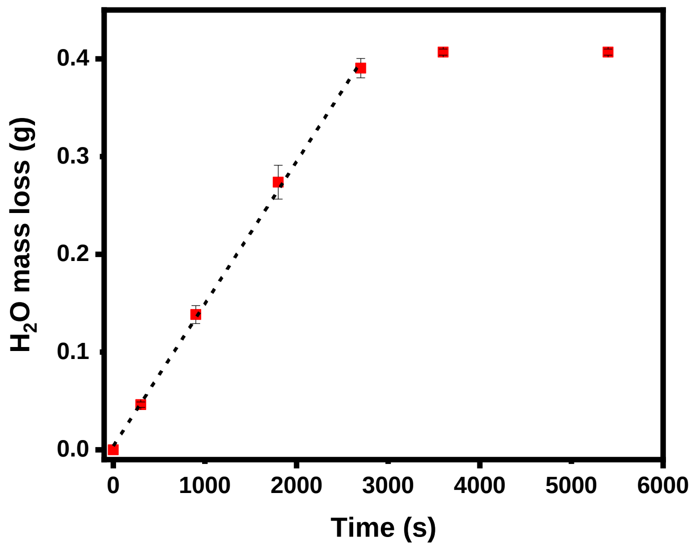

Figure S4. Mass loss during film formation at fast evaporation conditions (see *Table 1* for conditions).

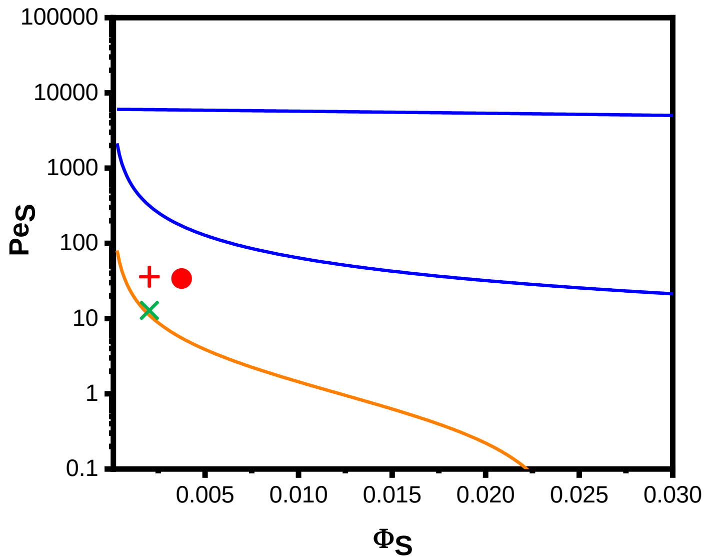

Figure S5. Péclet number (Pe_S) and volume fraction of small particles (ϕ_s) state diagram which illustrates the stratification of binary colloidal mixtures obtained from the Schulz-Sear model (blue line) or ZJD model (orange line). The area between the two blue lines, or above the orange line corresponds to where small-on-top stratification is predicted to occur according to each of the models. The points represent fast (red) or slow (green) evaporation rates, and the two TiO_2 volume fractions used for the same-charge systems as mentioned in *Table 1*. Experimental observations for stratification are indicated by symbols: (+) for small-on-top, (×) for no stratification, or (●) for an intermediate situation, e.g., sandwich structure.

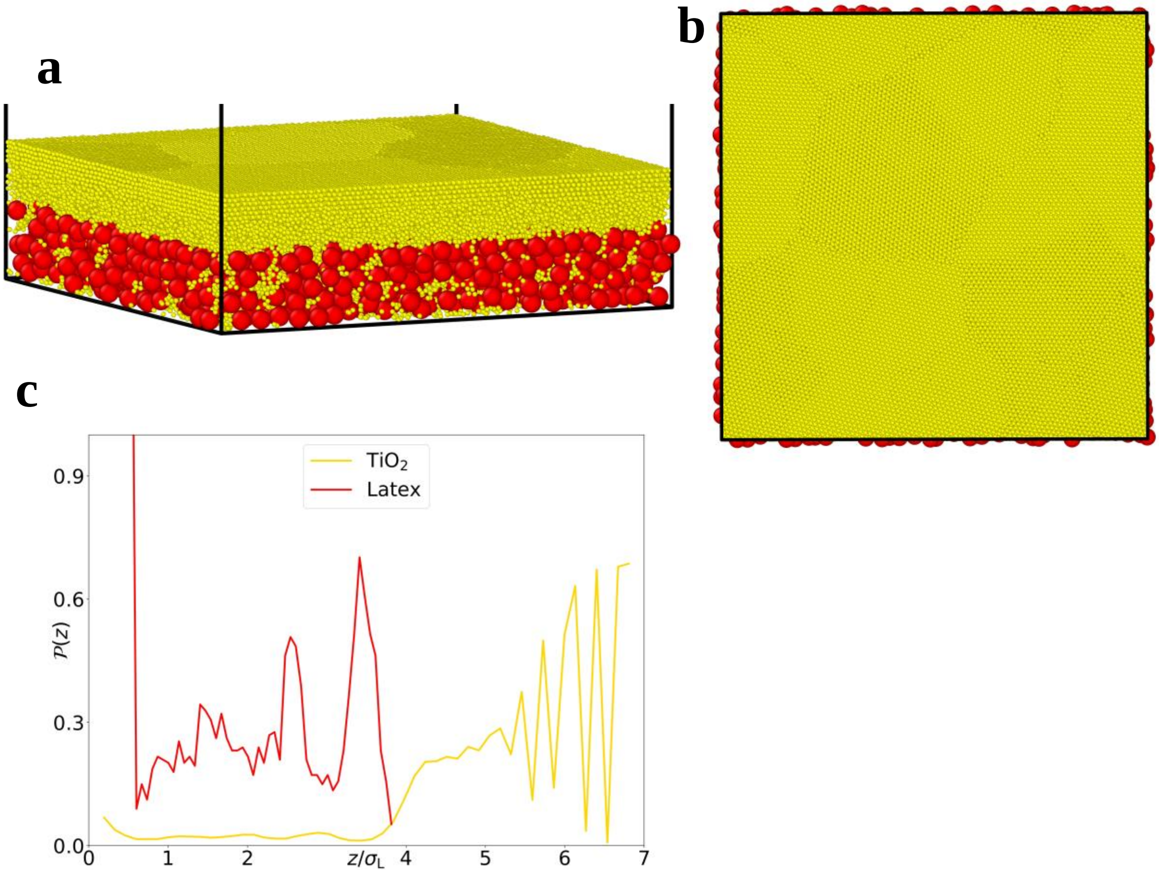

Figure S6. Brownian dynamics simulations to model the $L+Ti_{50,fast}$ system. Snapshots of the films' (a) cross-section and (b) top surface taken at the end of the drying process, along with (c) particle probability distributions perpendicular to the film surface. Small (TiO_2) and large (latex) particles are depicted in yellow and red, respectively.

Table S1. Raw data spreadsheet obtained from testing the photocatalytic activity of films against MRSA bacteria (following ISO 27447:2009).

	**Dil.**[a)]	**Rep**[b)] **1**	**Rep 2**	**Rep 3**	**Rep Ave.**[d)]	**Std. Er.**[e)]	**% Er.**[f)]	**on chip**[g)] **x10**^{5}	**log10**	**log*Red***[h)]	**Log*Red* UV or dark**[i)]	**% Er.**	**Photoc. Act.**[j)]	**% Er.**
**Control T=0**	-1	TNTC[c)]	TNTC	TNTC										
	-2	306	320	293	306.3	11.0		3.06	5.49					
	-3	28	32	40										
	-4	5	2	2										
**Control T=4h_UV**	-1	TNTC	TNTC	TNTC										
	-2	340	226	313	293.0	48.6	0.17	2.93	5.47	0.02				
	-3	30	21	33										
	-4	2	2	2										
**L+Ti$_{30,fast}$ T=4h_UV**	-1	TNTC	TNTC	TNTC										
	-2	47	102	83	77.3	22.8	0.29	0.77	4.89	0.60	0.58	0.23	0.51	0.21
	-3	9	6	7										
	-4	18	0	3										
**L-Ti$_{30,slow}$ T=4h_UV**	-1	241	188	317										
	-2	19	11	39	23.0	11.8	0.51	0.23	4.36	1.12	1.11	0.34	0.30	0.30
	-3	0	3	3										
	-4	1	0	0										
**Control T=4h_Dark**	-1	TNTC	TNTC	TNTC										
	-2	302	235	213	250.0	37.9	0.15	2.50	5.40	0.09				
	-3	27	19	32										
	-4	2	1	1										

**L+Ti$_{30,fast}$ T=4h_Dark**	-1	TNTC	TNTC	TNTC										
	-2	163	207	273	214.3	45.2	0.21	2.14	5.33	0.16	0.07	0.18		
	-3	24	20	23										
	-4	2	0	1										
**L-Ti$_{30,slow}$ T=4h_Dark**	-1	315	111	TNTC										
	-2	39	-	67	39.0	14.0	0.36	0.39	4.59	0.90	0.81	0.26		
	-3	4	-	6										
	-4	0	0	0										
**Control T=0**	-1	TNTC	TNTC	TNTC										
	-2	205	274	260	246.3	29.8		2.46	5.39					
	-3	32	35	23										
	-4	3	2	1										
**Control T=4h_UV**	-1	TNTC	TNTC	TNTC										
	-2	163	164	169	165.3	2.62	0.02	1.65	5.22	0.17				
	-3	23	23	17										
	-4	0	0	0										
**L+Ti$_{50,fast}$ T=4h_UV**	-1	247	TNTC	TNTC										
	-2	25	-	27	26.0	1.00	0.04	0.26	4.41	0.98	0.80	0.03	0.12	0.04
	-3	1	-	2										
	-4	0	0	1										
**L-Ti$_{30,fast}$ T=4h_UV**	-1	232	296	232										
	-2	13	23	18	18.0	4.08	0.23	0.18	4.26	1.14	0.96	0.12	0.28	0.14
	-3	2	4	0										
	-4	0	0	0										

**Control T=4h_Dark**	-1	TNTC	TNTC	TNTC										
	-2	228	260	236	241.3	13.6	0.06	2.41	5.38	0.01				
	-3	31	28	27										
	-4	2	0	7										
**L+Ti$_{50,fast}$ T=4h_Dark**	-1	236	TNTC	TNTC										
	-2	-	54	47	50.5	3.50	0.07	0.51	4.70	0.69	0.68	0.06		
	-3	5	9	3										
	-4	0	2	0										
**L-Ti$_{30,fast}$ T=4h_Dark**	-1	TNTC	132	TNTC										
	-2	36	-	64	50.0	14.0	0.28	0.5	4.70	0.69	0.68	0.17		
	-3	9	0	4										
	-4	0	0	0										

[a)]serial dilutions (log dilutions) of initial recovery solution; [b)]number of repetition; [c)]stands for too numerous to count, where bacterial colonies were more than 350; [d)]average between Rep 1,2,3; [e)]standard deviation; [f)]Rep Ave. / Std. Er; [g)]number of bacterial colonies on the film. Calculated by Rep Aver. × dil. Number × 0.1 × 100 µl; [h)]log reduction of MRSA colonies, obtained after subtracting the number of viable colonies on control (non-treated coverslip) just after inoculation; [i)]log reduction of MRSA colonies after subtracting the number of viable colonies on control (non-treated coverslip) after UVA irradiation for 4 h, or in the dark for 4 h respectively; [j)]photocatalytic activity, calculated by Log*Red* UV – Log*Red* Dark.

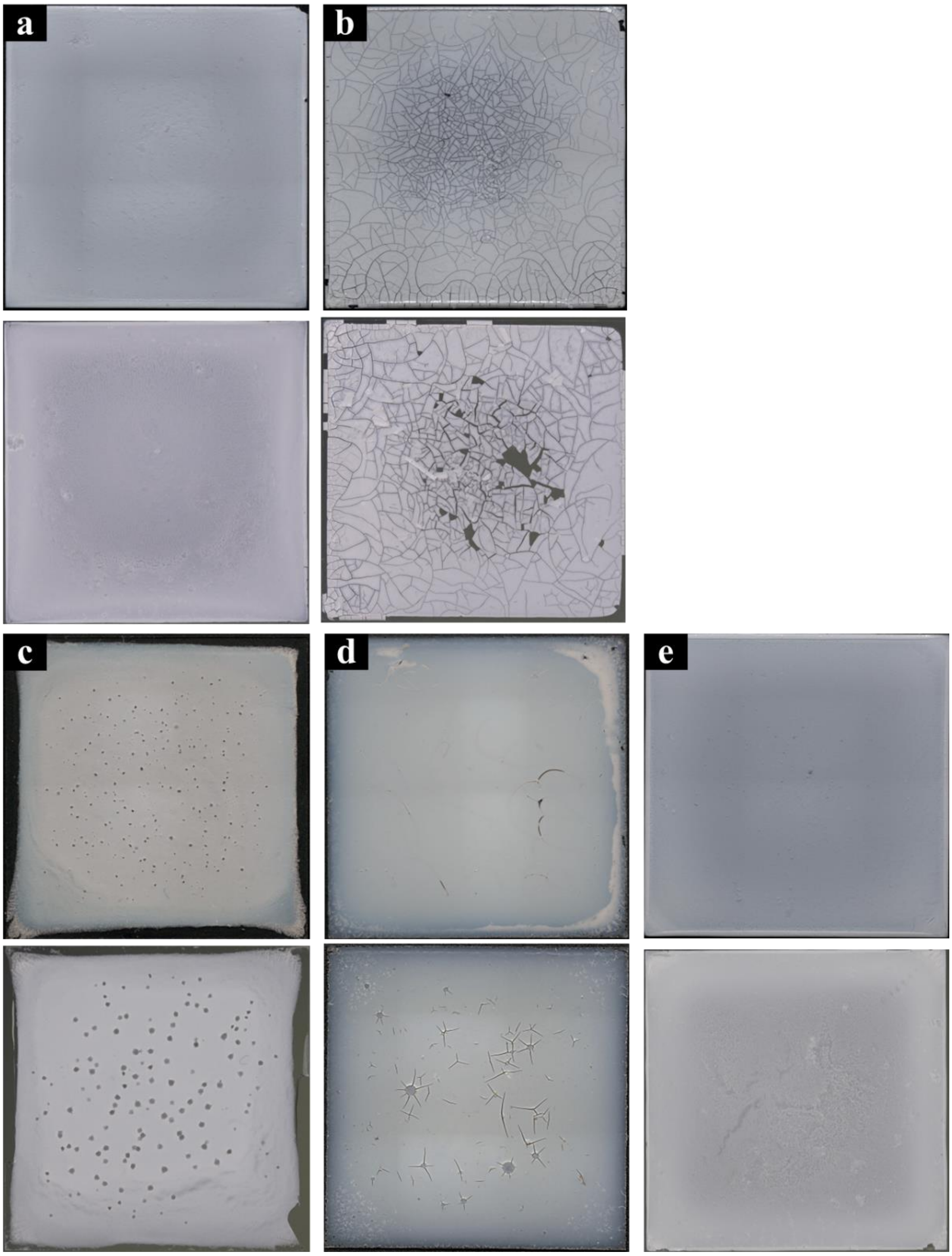

Figure S7. Optical microscope images of the five systems on glass substrates (18 × 18 mm^2): (a) L+Ti$_{30,fast}$, (b) L+Ti$_{30,slow}$, (c) L-Ti$_{30,fast}$, (d) L-Ti$_{30,slow}$ and (e) L+Ti$_{50,fast}$ as prepared and dried (top), and after 15 min soaking in isopropyl alcohol (bottom).

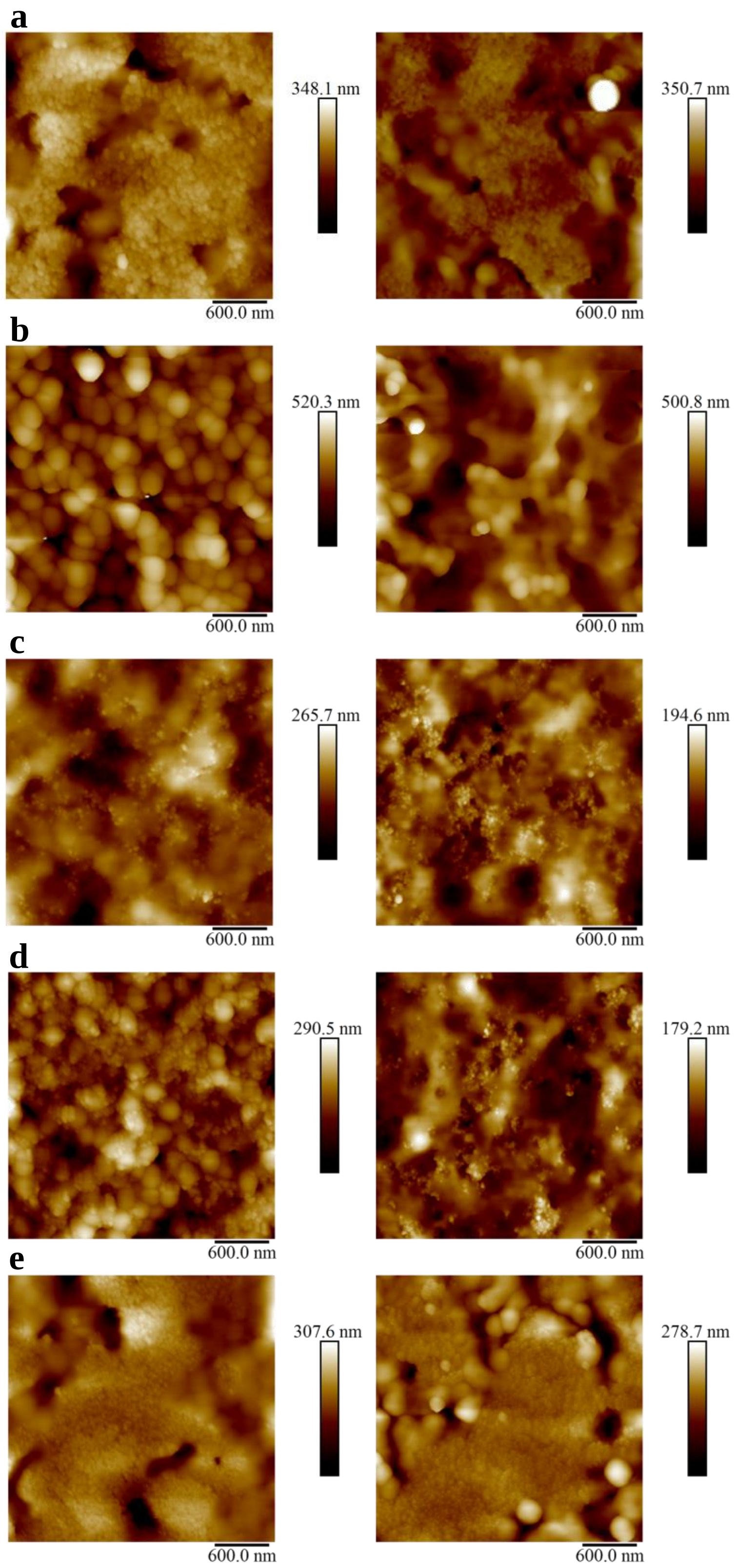

Figure S8. 3 × 3 μm^2 AFM topography images of the top surface of the films (a) L+Ti$_{30,fast}$, (b) L+Ti$_{30,slow}$, (c) L-Ti$_{30,fast}$, (d) L-Ti$_{30,slow}$, and (e) L+Ti$_{50,fast}$ taken after soaking the films in water for 24 h (left) or 70% isopropyl alcohol for 15 min (right).

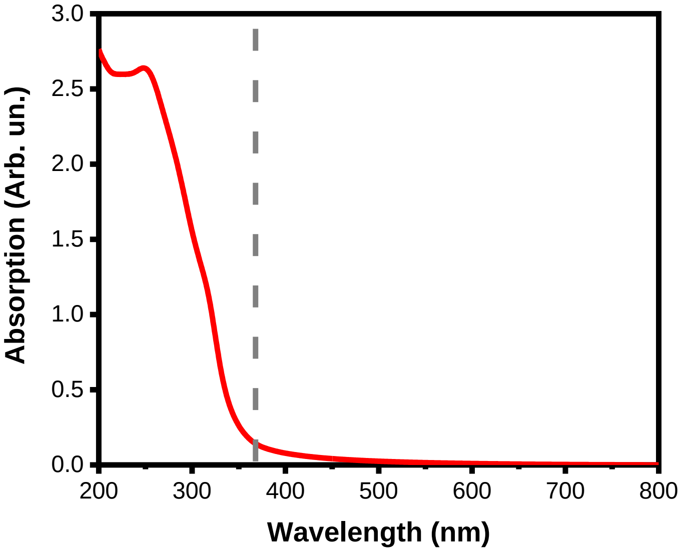

Figure S9. UV-vis absorption spectrum of 0.1 wt.% TiO_2 nanoparticle dispersion in water (pH = 3-3.5), prepared as described in the experimental section. Dashed line corresponds to the wavelength used in the antibacterial tests (368 nm).

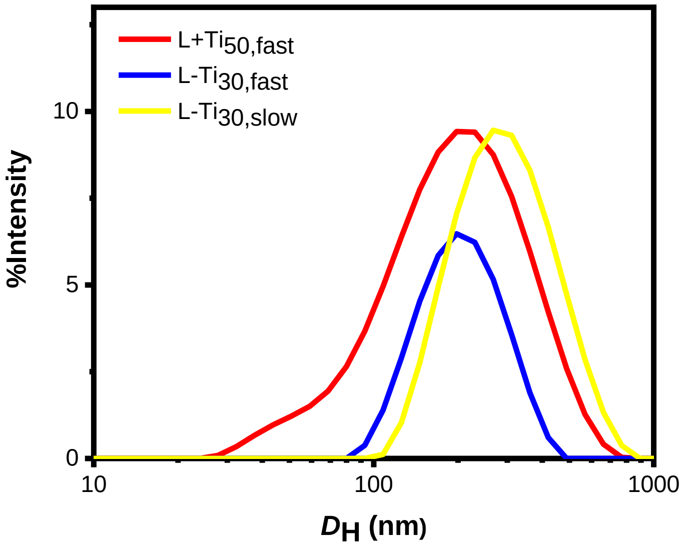

Figure S10. Particle size distribution measured by dynamic light scattering (DLS) of water retrieved from the surface of L+$Ti_{50,fast}$, L-$Ti_{30,fast}$, and L-$Ti_{30,slow}$ films after 4h of soaking.

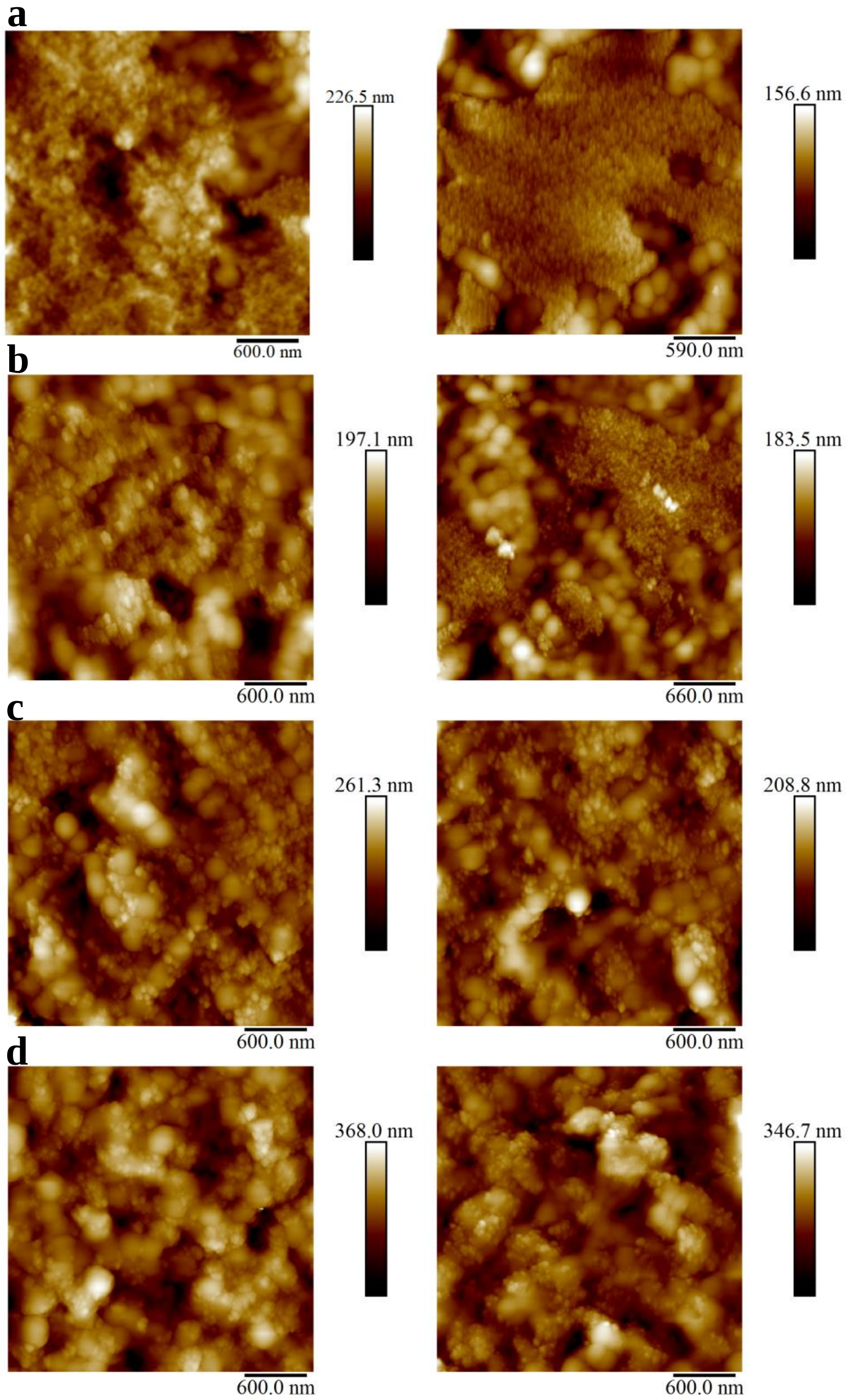

Figure S11. 3 × 3 μm^2 AFM topography images of the top surface of the films L+Ti$_{50,fast}$ (a), L+Ti$_{30,fast}$ (b), L-Ti$_{30,fast}$ (c), and L-Ti$_{30,slow}$ (d) taken after antibacterial testing, and soaking in 70% isopropyl alcohol for 15 min.

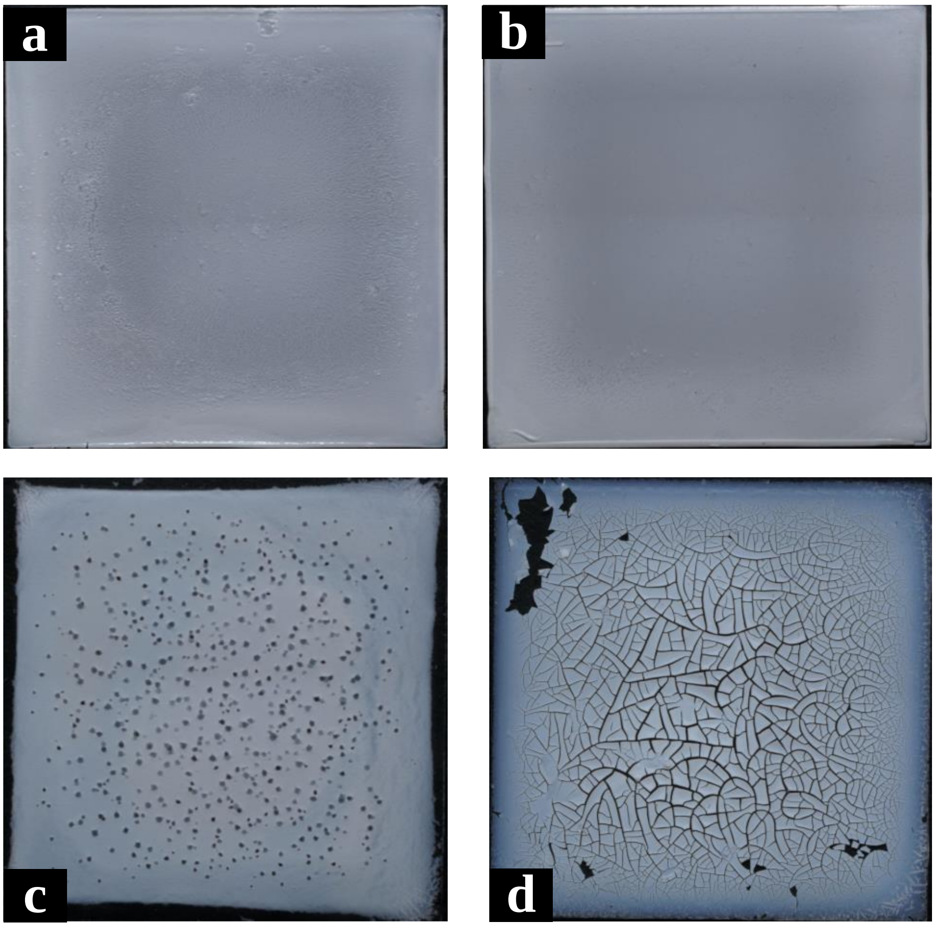

Figure S12 – Optical microscope images of the films L+Ti$_{50,fast}$ (a), L+Ti$_{30,fast}$ (b), L-Ti$_{30,fast}$ (c), and L-Ti$_{30,slow}$ (d) taken after antibacterial testing, and soaking in 70% isopropyl alcohol for 15 min.

Contact angle measurements

Table S2. Water contact angle data.

Sample	UV exposure	Contact angle (°)
L+Ti$_{50,fast}$	No	79 ± 4
L+Ti$_{50,fast}$	Yes	74 ± 4
L+Ti$_{30,fast}$	No	73 ± 1
L+Ti$_{30,fast}$	Yes	75 ± 3
L-Ti$_{30,fast}$	No	89 ± 2
L-Ti$_{30,fast}$	Yes	96 ± 1
L-Ti$_{30,slow}$	No	93 ± 2
L-Ti$_{30,slow}$	Yes	109 ± 4

References

(1) Velasquez, E.; Rieger, J.; Stoffelbach, F.; D'Agosto, F.; Lansalot, M.; Dufils, P. E.; Vinas, J. Surfactant-Free Poly(Vinylidene Chloride) Latexes via One-Pot RAFT-Mediated Aqueous Polymerization. *Polymer (Guildf).* **2016**, *106*, 275–284. https://doi.org/10.1016/j.polymer.2016.08.083.

(2) Engström, J.; Benselfelt, T.; Wågberg, L.; D'Agosto, F.; Lansalot, M.; Carlmark, A.; Malmström, E. Tailoring Adhesion of Anionic Surfaces Using Cationic PISA-Latexes-towards Tough Nanocellulose Materials in the Wet State. *Nanoscale* **2019**, *11* (10), 4287–4302. https://doi.org/10.1039/c8nr08057g.

(3) Utgenannt, A.; Maspero, R.; Fortini, A.; Turner, R.; Florescu, M.; Jeynes, C.; Kanaras, A. G.; Muskens, O. L.; Sear, R. P.; Keddie, J. L. Fast Assembly of Gold Nanoparticles in Large-Area 2D Nanogrids Using a One-Step, Near-Infrared Radiation-Assisted Evaporation Process. *ACS Nano* **2016**, *10* (2), 2232–2242. https://doi.org/10.1021/acsnano.5b06886.
